# Recent advances and prospects in the organocatalytic synthesis of quinazolinones

**DOI:** 10.3389/fchem.2022.991026

**Published:** 2022-09-14

**Authors:** Biplob Borah, Sidhartha Swain, Mihir Patat, L. Raju Chowhan

**Affiliations:** School of Applied Material Sciences, Centre for Applied Chemistry, Central University of Gujarat, Gandhinagar, India

**Keywords:** quinazolinone, organocatalyst, heterocycles, natural product, therapeutic agents

## Abstract

Quinazolinone, a bicyclic compound, comprises a pyrimidine ring fused at 4´ and 8´ positions with a benzene ring and constitutes a substantial class of nitrogen-containing heterocyclic compounds on account of their frequent existence in the key fragments of many natural alkaloids and pharmaceutically active components. Consequently, tremendous efforts have been subjected to the elegant construction of these compounds and have recently received immense interest in synthetic and medicinal chemistry. The domain of synthetic organic chemistry has grown significantly over the past few decades for the construction of highly functionalized therapeutically potential complex molecular structures with the aid of small organic molecules by replacing transition-metal catalysis. The rapid access to this heterocycle by means of organocatalytic strategy has provided new alternatives from the viewpoint of synthetic and green chemistry. In this review article, we have demonstrated a clear presentation of the recent organocatalytic synthesis of quinazolinones of potential therapeutic interests and covered the literature from 2015 to date. In addition to these, a clear presentation and understanding of the mechanistic aspects, features, and limitations of the developed reaction methodologies have been highlighted.

## Introduction

**FIGURE 1:**
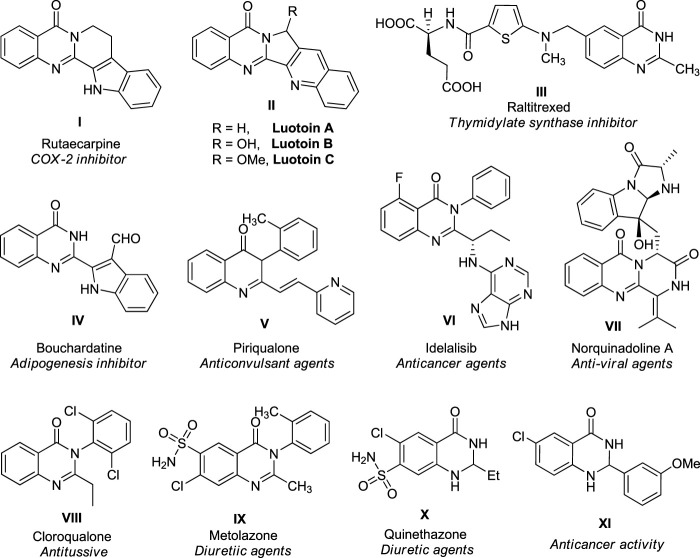
Representative examples of natural products and therapeutically potential molecules.

Heterocyclic compounds are among the most versatile organic compounds in natural and biologically active synthetic materials, pharmaceuticals, and synthetic intermediates (Bur and Padwa, 2004; Dwivedi et al., 2020; Borah et al., 2021a; Borah and Chowhan, 2021; Borah and Chowhan, 2022a; Kumar B. et al., 2022). The basic unit of heterocycles comprises a ringed structure with a heteroatom like nitrogen, oxygen, or sulfur, other than carbon, and the properties of the compound depend significantly on the number and types of heteroatoms present in the ring. Owing to their immense importance in a variety of disciplines of medicinal chemistry, drug design, and functional materials, the construction of heterocyclic compounds, especially those containing nitrogen and oxygen, has received enormous interest in organic synthesis (Majumdar et al., 2014; Patrusheva et al., 2018; Chugh et al., 2020; Anantha et al., 2021; Mermer et al., 2021; Xing et al., 2021; Borah et al., 2022a). Among various nitrogen-containing heterocycles, quinazolinone, a bicyclic compound with a pyrimidine system fused at 4**´** and 8**´** positions with a benzene ring, has recently received a great deal of attention in organic and medicinal chemistry (Auti et al., 2020; Khan et al., 2014; Kshirsagar, 2015; Dherbassy et al., 2021; Hao et al., 2021). Quinazolinone and its derivatives are an essential heterocyclic skeleton that serves a prominent role in several cellular processes and have been established for their remarkable therapeutic significance as antihypertensive, antimicrobial, antihyperlipidemic, anti-inflammatory, and anticonvulsant activities (Mhaske and Argade, 2006; Khan et al., 2016). At the same time, some of their derivatives are also used as antitumor chemotherapeutic agents (Hekal and Abu El-Azm, 2018; Hakim et al., 2022). For instance, some representative examples of therapeutically potential quinazolinone derivatives are described in Figure 1. The natural alkaloid rutaecarpine isolated from the unripe fruit of *Evodia rutaecarpa* was found to possess anti-inflammatory properties as it inhibits cyclooxygenase 2 (Chen and Chen, 1933; Bergman and Bergman, 1985; Moon et al., 1999). Quinazolinone containing another natural alkaloid, luotonin A, has been derived from *Peganum nigellastrum*, a Chinese herbal medicinal plant, and demonstrated to exhibit cytotoxic effects against murine leukemia P388 cell line (Ma et al., 1997) and recognized as antiviral and antiphytopathogenic fungus agents (Hao et al., 2020). Raltitrexed (Gunasekara and Faulds, 1998) from the quinazolinone family under the brand name Tomudex^®^) is constructed as a particular thymidylate synthase inhibitor and used as a chemotherapeutic agent against colorectal cancer (Köhne et al., 1998). Bouchardatine, a β-indoloquinazoline alkaloid isolated from the aerial part of *Bouchardatia neurococca,* has been recognized as an adipogenesis inhibitor (Rao et al., 2015). The quinazolinone drug idelalisib, commercially available as Zydelig, was used for the treatment of chronic lymphocytic leukemia (Shanafelt et al., 2015; Zirlik and Veelken, 2018). Norquinadoline A isolated from *Cladosporium* sp. PJX-41, a mangrove-based fungus, was known to have antiviral activity (Peng et al., 2013). In addition to these, a diverse range of natural and non-natural quinazolinone products displayed remarkable biological activities such as anticonvulsant, antitussive, antidiabetic, diuretic, hypnotic, sedative, analgesic, and many more (Gupta et al., 2018).

**SCHEME 1:**
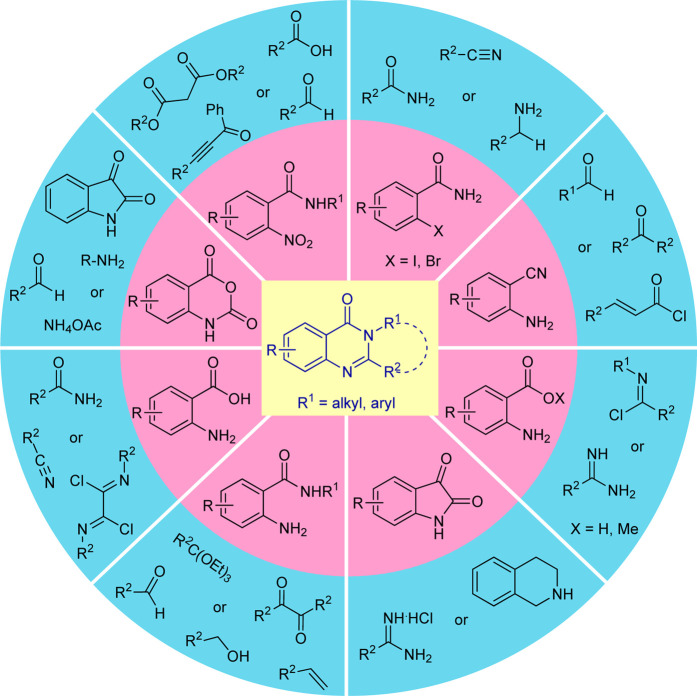
Various starting materials used for the synthesis of quinazolinones.

Recognizing such promising features, elegant synthetic routes for the construction of these heterocycles, starting from the traditional method to the green catalytic one, have been developed over the past decades (Scheme 1). Although the developed methodologies were well established, sometimes they suffer several serious drawbacks such as the utilization of hazardous reagents, transition-metal catalysts, toxic volatile solvents, high temperatures, harsh reaction conditions, long reaction times, and excessive energy inputs that have a negative impact on both the environment and the overall chemical process, making it difficult to create eco-friendly and sustainable nature. Consequently, significant efforts have been dedicated in the last decades to overcoming these limitations by improving the reaction conditions for the synthesis of these heterocycles that are of significant interest to the scientific community.

Over the last decades, the field of organic synthesis has been mainly dominated by metal catalysis (Nakamura and Yamamoto, 2004; D'Souza and Mueller, 2007; Shang and Liu, 2013) and biocatalysis (Sugai et al., 1997; Reetz, 2013). Despite being powerful catalytic systems that have already been successfully employed for the assembly of quinazolinones and have huge applications in numerous organic syntheses to build valuable structural frameworks such as drug molecules and natural product analogs, transition metal catalysts have many limitations and drawbacks from the perspectives of synthetic efficiency and green chemistry (Chatterjee et al., 2011; Zhou 2016). Intriguingly, it is highly desired to develop a chemical process that uses alternative materials for synthetic purposes that are not only environmentally friendly but also easily available in bulk quantities anywhere at a very low price for the synthesis of complex molecules of potential therapeutic significance with high atom- and step-economies by reducing or avoiding the utilization of transition metal catalysts, co-catalyst, or any additives (Bhunia et al., 2012; Sun and Shi, 2014; Poudel et al., 2018; Khan et al., 2020; Borah and Chowhan, 2022b).

In this regard, the use of small organic molecules called organocatalysts has received substantial attention in organic transformation due to their remarkable properties (List, 2007; Bertelsen and Jørgensen, 2009; Borah et al., 2021b). Not only metal catalysts but also organocatalysts are better replacements for the highly substrate-specific and extremely sensitive biocatalyst. The existence of organocatalyst has led to a revolution in the synthesis of molecular diversity and complexity in an asymmetric and non-asymmetric manner *via* several activation modes and has turned into one of the most important hot topics of current research in terms of synthetic efficiency and from the green chemistry point of view (Dalko and Moisan, 2001; Barbas, 2008; MacMillan, 2008). The distinctive ability to accomplish chemical transformation through different activation modes, avoidance of expensive catalysts and metal catalyst(s), high stability, ready availability, easy recoverability, lower activation energy, high efficiency, and an immediate reduction in the toxicity and reaction costs offers organocatalytic synthetic approaches as efficient routes for the synthesis of a diverse range of quinazolinone scaffolds from the green and sustainable chemistry viewpoints (Renzi and Bella, 2012; Ren and Wang, 2013; Borah et al., 2021d).

Several review articles focus on the synthesis of quinazolinones from non-conventional to conventional processes (Khan et al., 2015; Khan et al., 2014; Rohokale and Kshirsagar, 2016; Faisal and Saeed, 2021). He et al. reviewed the synthesis of quinazolinone derivatives based on different types of reactions used (He et al., 2014) Abbas et al., and demonstrated the utilization of isatoic anhydride in the synthesis of quinazolinones (Abbas et al., 2016). Maiden and Harrity disclosed the metal-catalyzed synthetic approaches to quinazolinones (Maiden and Harrity, 2016). Heravi and Mohammadkhani reviewed the microwave-assisted synthesis of quinazoline and quinazolinones (Mohammadkhani and Heravi, 2020). Kumar et al. summarize the synthesis of quinazolinones by using different nanoparticles as catalytic systems (Kumar P. et al., 2022). Here, we have demonstrated a recent outlook on the synthesis of quinazolinones by using various organocatalytic systems and cover the report from 2015 to 2022. The mechanistic rationalizations, remarkable advantages, limitations, and scopes of future exploration have also been discussed. The review has been organized based on the types of substrates and catalysts employed.

## Synthesis of quinazolinones from two-component organocatalytic reactions

### Acid-catalyzed two-component synthesis of quinazolinones

**SCHEME 2:**
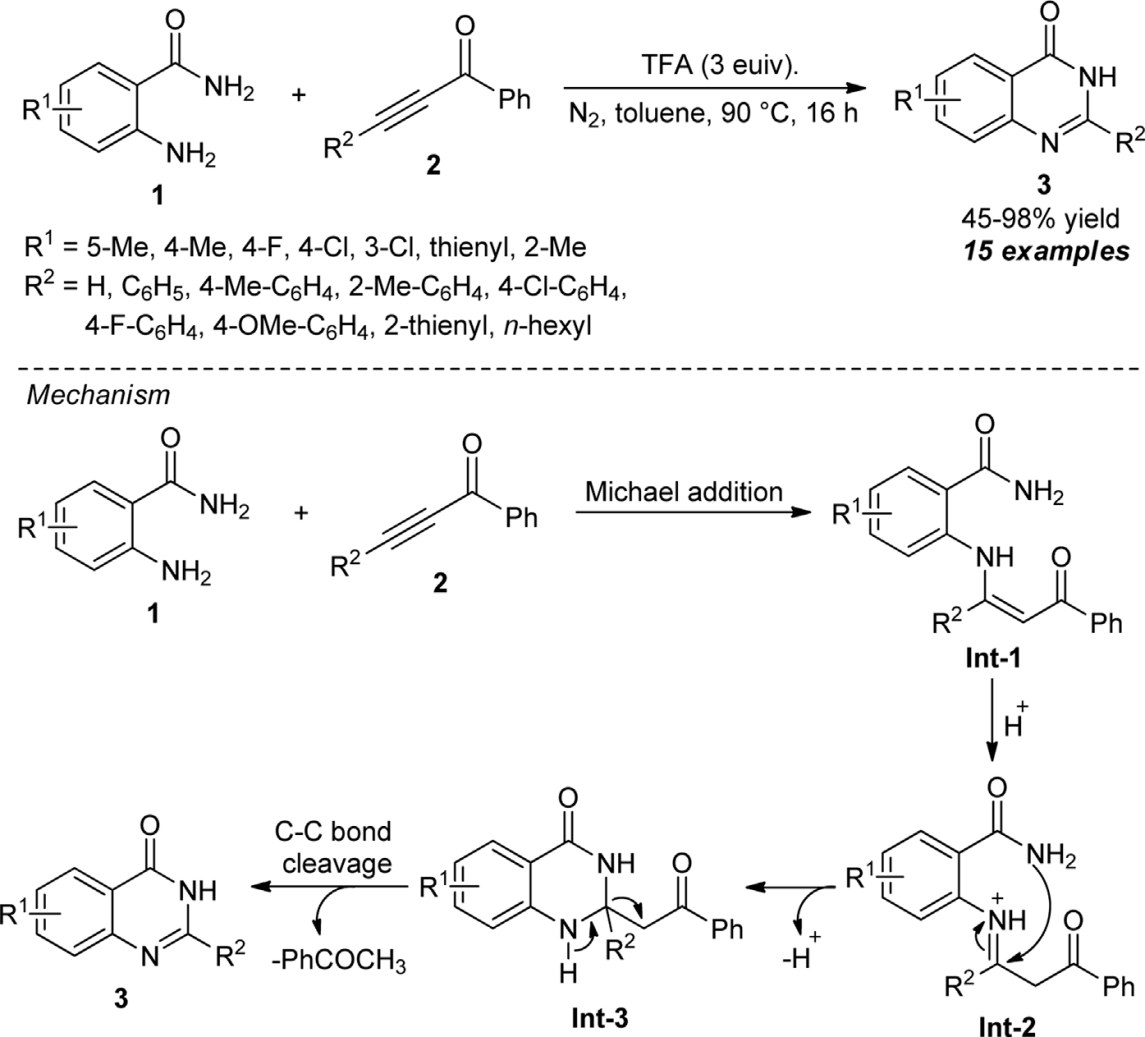
TFA-promoted C-C triple bond cleavage strategy to access quinazolinones.

In 2015, Yang *et al.* devised a highly convenient metal-free approach for the assembly of 2-aryl (alkyl)-quinazolin-4(3*H*)-ones **3** *via* selective breaking of the C-C triple bond of ketoalkynes **2**. Under oxidant-free conditions, the trifluoroacetic acid (TFA)-promoted reactions of various anthranilamides **1** and ketoalkynes **2** furnished the representative products in 45–98% yields (Scheme 2) (Yang et al., 2015). The reaction was applicable to aryl- and alkyl-substituted ketoalkynes, and the implication of steric and electronic effects of various substituents on the reaction is minimal. Despite having these successful attempts, they further investigated the reaction with various anthranilamides possessing electron-deficient and electron-rich groups, and in all the cases, the products were found to be formed in quantitative yields. The overall process for this transformation starts with the TFA-mediated Michael addition of **1** and **2** to form the enaminone intermediate **Int-1**. The subsequent intramolecular cyclization of **Int-1** afforded **Int-3,** which is followed by cleavage of the C-C triple bond to generate the final products **3**.

**SCHEME 3:**
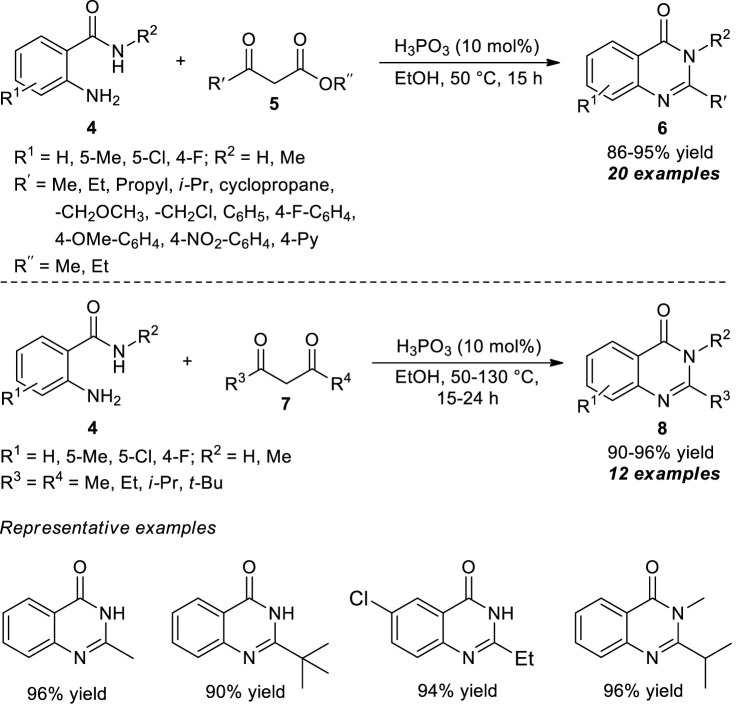
H_3_PO_3_ catalyzed rapid synthesis of quinazolinones *via* selective C-C bond cleavage strategy.

The selective C-C bond cleavage strategy under metal-free conditions was also developed by Li et al. at the same time. With the aid of phosphorous acid (H_3_PO_3_), the representative quinazolinone products **6** derived from the cyclo-condensation of 2-aminobenzamides **4** and β-ketoesters **5** have been accomplished in 86–95% yields. Introducing various aprotic solvents such as ethanol (EtOH) and methanol (MeOH) offered an excellent yield of products as compared to an aprotic solvent such as dimethylformamide (DMF), toluene, acetonitrile (CH_3_CN), and dioxane that provided products with lower yields. Furthermore, the yield of the product was increased with reduced reaction time by increasing the temperature from 25 to 50 °C (Scheme 3) (Li et al., 2015). Regardless of the steric hindrance and electronic effect of the substituents, this acid-catalyzed cyclo-condensation could be employed in a variety of β-ketoesters to yield the corresponding quinazolinones in excellent yields. To broaden the scope of the present protocol, the reaction with β-diketones **7** was also explored. Despite demonstrating a broad range of aliphatic diketones, the steric effect plays a crucial role, and bulky substitutions such as aryl-substituted β-diketones were inactive in cyclo-condensation reactions, which marks the limitations of the present approach.

**SCHEME 4:**
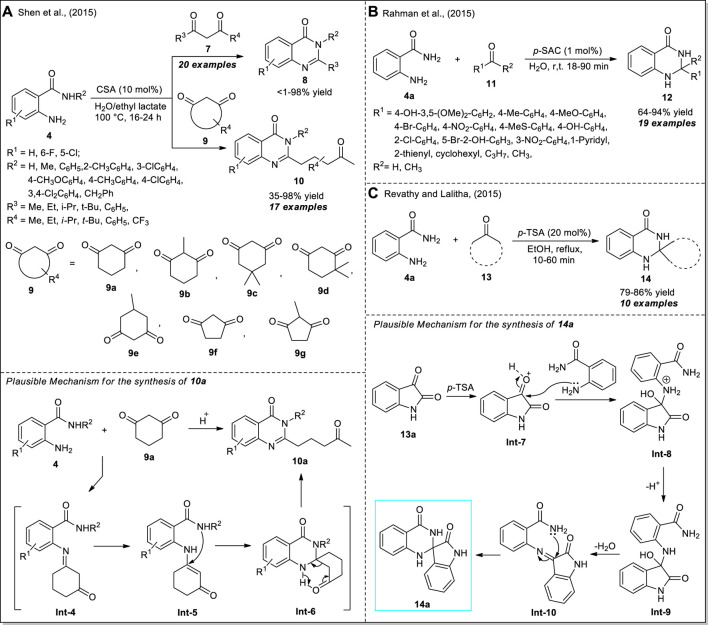
CSA, *p*-SAC, and *p*-TSA catalyzed one-pot synthesis of quinazolinones **8** & **10** **(A)**, dihydro‐quinazolinones **12** **(B)**, and spiro-quinazolinones **14** **(C)**, respectively.

Inspired by the elegant advantages of the previous work, Shen et al. devised a green and straightforward technique for the selective C-C bond cleavage of acyclic or cyclic 1,3-diketones **7** or **9** to access a wide variety of 4(3*H*)-quinazolinones (Scheme 4A) (Shen et al., 2015). By using 10 mol% of camphorsulphonic acid (CSA) as a Brønsted acid catalyst in an aqueous solution of biodegradable ethyl lactate, the corresponding products **8** and **10** derived from the reaction of 2-aminobenzamides **4** and diketones **7** or **9** have been achieved in <1–98% and 35–98% yield, respectively. The execution of the reaction with various substitutions on **4** or **7** or **9** was found to have no greater influence on the yield of the products. However, the sterically hindered bulky substituents such as the *tert*-Butyl group present on 1,3-diketone **7** significantly decreased the yield of the product (<1%), whereas smaller groups such as methyl yielded 98% of the product. The mechanism for the formation of **10a** begins with the generation of **Int-4** from the condensation of **4** and **9a**, which was further tautomerized to **Int-5**. Subsequently, the intramolecular cyclization of **Int-5** and final C-C bond cleavage occurred to form the product **10a**.

Rahman et al. demonstrated the catalytic activity of *p*-sulfonic acid calix [4] arene (*p*-SAC) as an organocatalyst in the efficient construction of 2,3-dihydroquinazolin-4(1*H*)-ones **12** from the direct cyclo-condensation of anthranilamide **4a** with aldehydes **11** (Scheme 4B) (Rahman et al., 2015). With the aid of only 1 mol% of the catalyst, along with water as the green solvent, the representative products **12** were accomplished in moderate to excellent yields at room temperature. The synthetic potentiality of the current strategy was established by recovering and reusing the catalyst for up to five successive sessions with a minimal change in catalytic property. The reaction conditions tolerate an extensive range of aryl-, heteroaryl-, and alkyl-substituted aldehydes. Moreover, the reaction is resistant to steric and electronic effects and provided significant yield regardless of aromatic, electron-withdrawing, or donating group. However, the reaction with acetone or propyl aldehydes required a longer reaction time to complete as compared to other aldehydes. In a similar manner, a very efficient *p*-toluene sulfonic acid (*p*-TSA) catalyzed one-pot approach for the assembly of spiro quinazolinones from the reaction of anthranilamide **4a** with ketones **13** was developed by Revathy and Lalitha (Scheme 4C) (Revathy and Lalitha, 2015). The reaction worked well with a wide range of ketones, and the representative products **14** were obtained in good to excellent yields within a short reaction duration. With various diketones as the substrate, the reactions with anthranilamide **4a** were completed in 1 h, whereas the same reaction with cyclic ketones was completed within 10 min. Furthermore, the utilization of 1,4-cyclohexanedione in a 1:1 ratio resulted in the formation of single spiro compounds on one side of the ketone; while using a 2:1 ratio, the dispiro compounds were formed. The reaction can proceed through the formation of imine intermediate **Int-10** *via* the addition of *p*-TSA activated **Int-7** with **4a**. The final cyclization of the -NH_2_ group on the double bond of imine of **Int-10** followed by the H^+^ shift afforded the product **14**.

**SCHEME 5:**
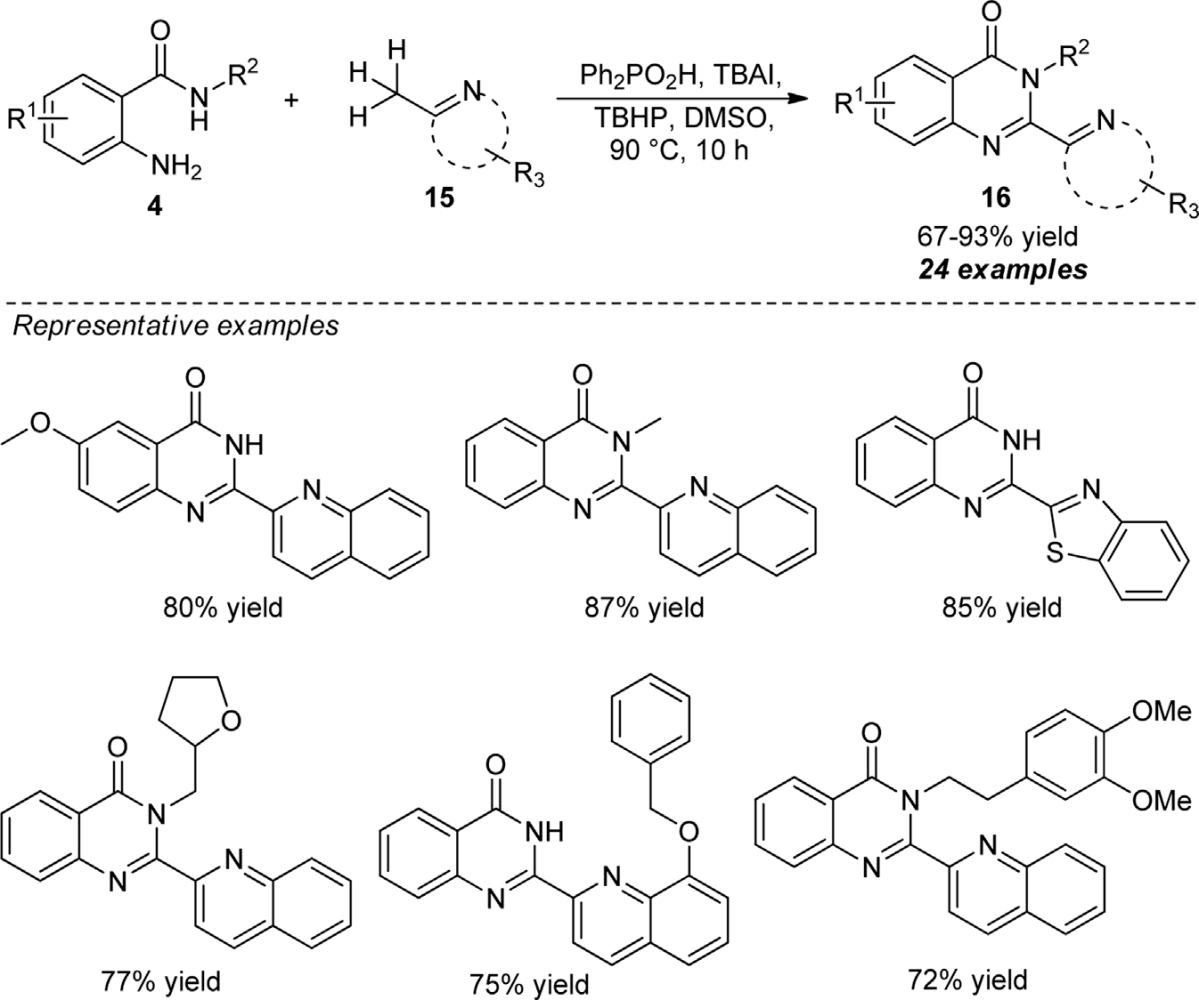
Acid catalyzed Csp^3^-H amination toward quinazolinones.

In 2016, Liu et al. disclosed a facile and straightforward approach for the elegant construction of 2-hetarylquinazolin-4(3*H*)-ones *via* metal-free oxidative amination of Csp^3^–H bonds. With the aid of diphenylphosphinic acid (Ph_2_PO_2_H) as the catalyst, tetra-*n*-Butyl ammonium iodide (TBAI) as the iodine source, and tert-butyl hydroperoxide (TBHP) as the oxidant, the representative products **16** derived from 2-aminobenzamides **4** with diverse electron-withdrawing or electron-donating groups and (2-aza-aryl)methanes **15** have been obtained in good to excellent yields (Scheme 5) (Liu et al., 2016). While substitutions on the *R* ^2^ position of **4** by groups like methyl smoothly reacted to afford the desired products, the presence of more hindered bulkier groups in the *R* ^2^ position decreased the yield of the products. On the other hand, diverse (2-aza-aryl) methanes such as 2-methylquinolines, 1-methylisoquinoline, 3-methylbenzo [*f*]quinolone, 2-methylquinoxaline, and 2-methylbenzo [*d*]thiazole worked well for this reaction. However, 2-methylnaphthalene and pyridine derivatives failed to yield any products under the standard reaction conditions, which marks the limitations of this chemistry and calls for further developments.

**SCHEME 6:**
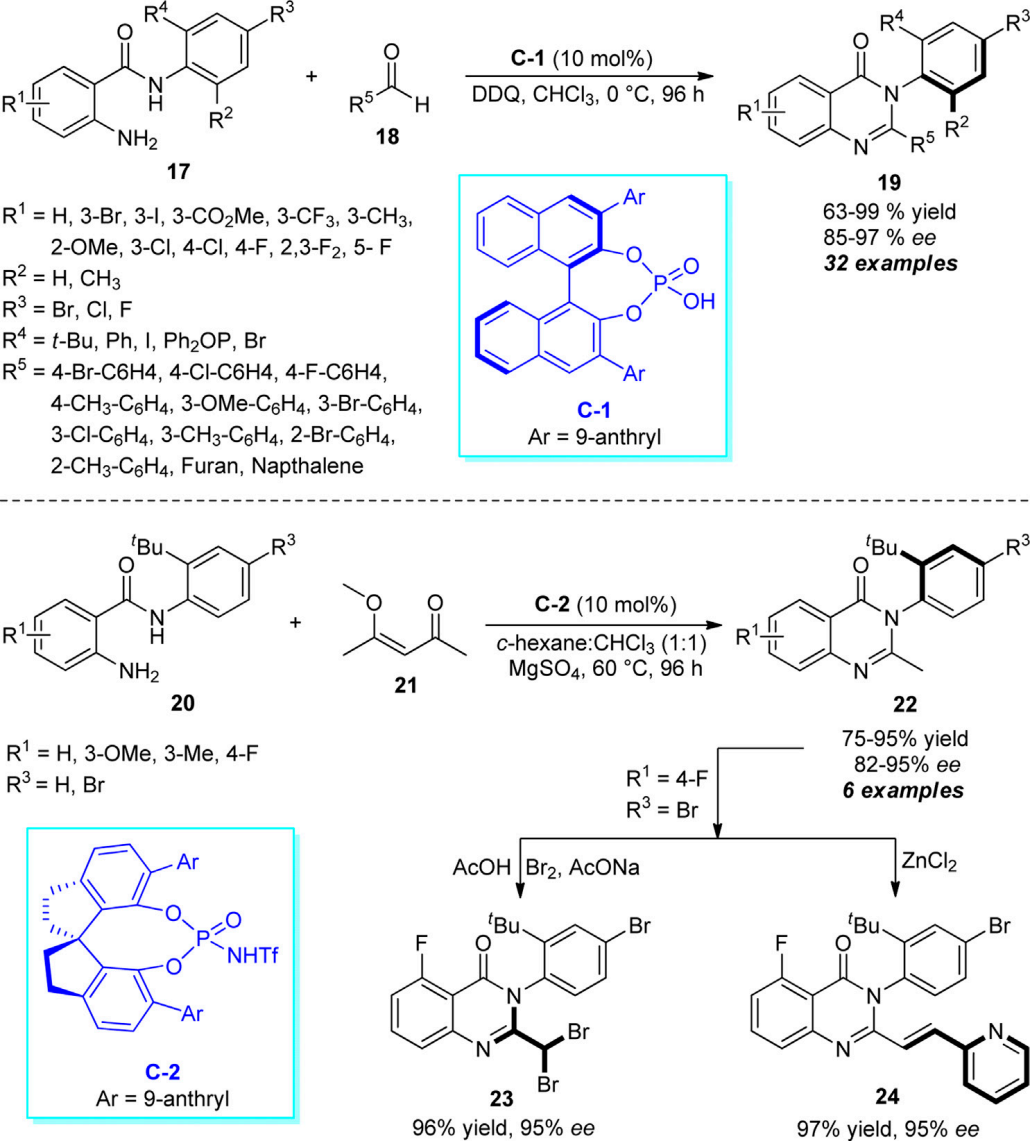
Enantioselective construction of axially chiral quinazolinones by chiral phosphoric acid catalysis.

A very simple and practical one-pot approach for the rapid access to enantiomerically pure aryl-quinazolinones catalyzed by chiral phosphoric acids was discovered by Wang et al. (Scheme 6) (Wang et al., 2017). With the aid of 10 mol% of **C-1** as the chiral catalyst and 2,3-dichloro-5,6-dicyano-1,4-benzoquinone (DDQ) as the oxidant, the treatment of 2-aminobenzamides **17** and aldehydes **18** was found to proceed at 0°C to furnish the axially chiral aryl quinazolinones **19** in moderate to excellent yields with good to excellent enantioselectivities. In addition to demonstrating the diverse substitutions on different positions of the *N*-aryl ring of 2-aminobenzamides **17**, a broad range of aryl and heteroaryl-substituted aldehydes possessing electron-deficient or electron-donating groups were established to be very efficient for this reaction. Furthermore, they also disclosed the *N*-triflylphosphoramides (**C-2**) catalyzed selective C-C bond cleavage strategy for the atroposelective construction of alkyl-substituted aryl quinazolinones **22**. Pleasingly, the reaction of *t-Bu* substituted 2-aminobenzamides **20** and 4-methoxypentenone **21** afforded the representative products **22** in 75–95% yields with good enantioselectivity. The synthetic efficiency of the protocol was further established by transforming the synthesized products into more complex products **23** and **24** without affecting the enantioselectivity.

**SCHEME 7:**
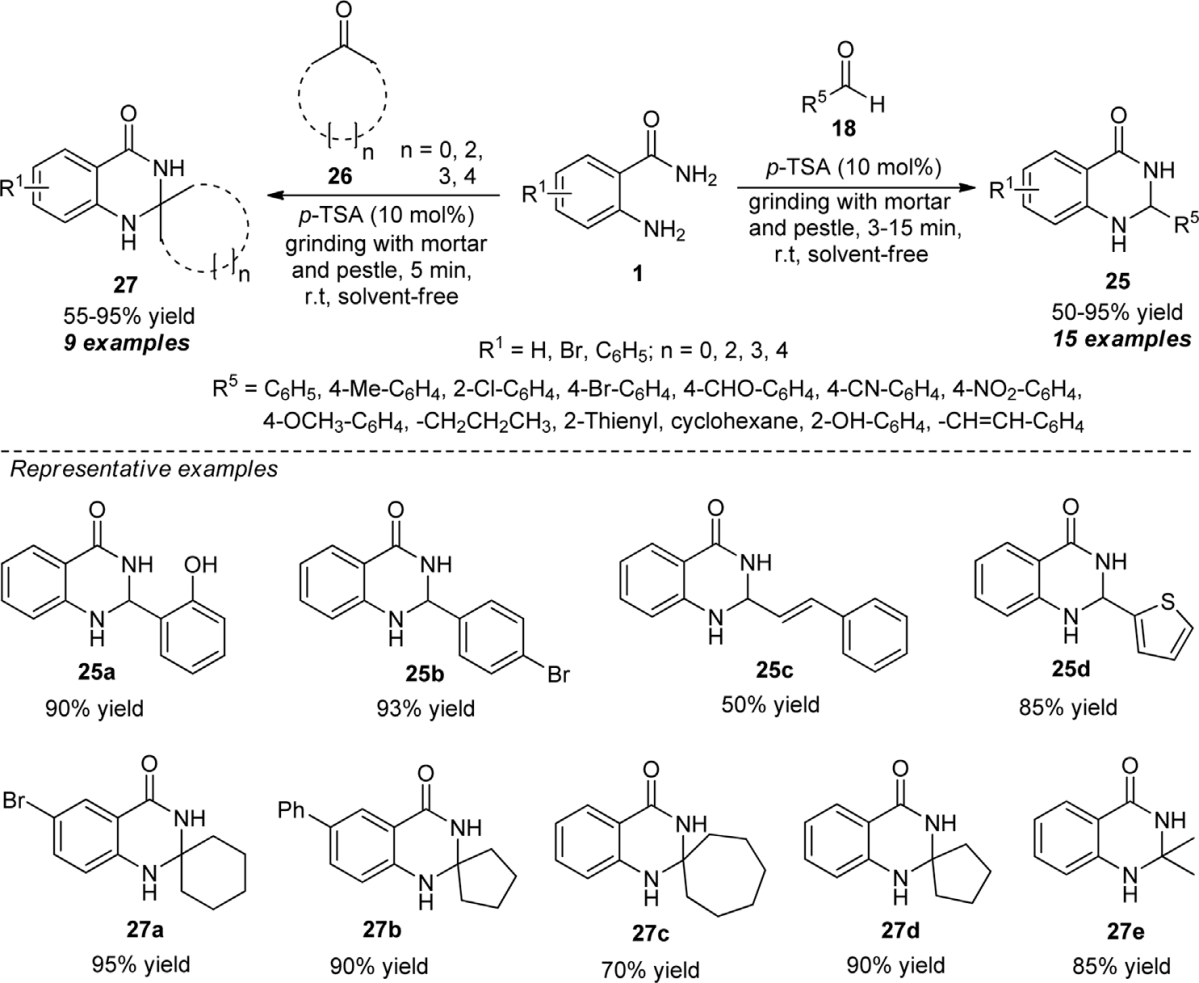
p-TSA catalyzed grinding-assisted synthesis of diverse quinazolinone derivatives.

Another achievement for the synthesis of different types of quinazolinones **25** and **27** has been accomplished by Yashwantrao et al. by employing mechanical activation as an environmentally benign approach (Scheme 7) (Yashwantrao et al., 2019). With the aid of 10 mol% of *p*-toluene sulfonic acid (*p*-TSA) as the Brønsted acid catalyst, the solvent-free reaction of anthranilamide **1** and aldehydes **18** under mechanochemical grinding conditions delivers the corresponding quinazolinone products **25** in moderate to excellent yield within 3–15 min. While the reaction of anthranilamide **1** with different carbonyl compounds **26** under the optimal condition delivers the quinazolinones of type **27** in moderate to excellent yield in only 5 min. Wide-range of functional group tolerances, mild reaction conditions, solvent-free, waste-free, and metal-free are some of the key advantages of this protocol. The practicality of the protocol was established by performing Gram scale synthesis in quantitative yield.

**SCHEME 8:**
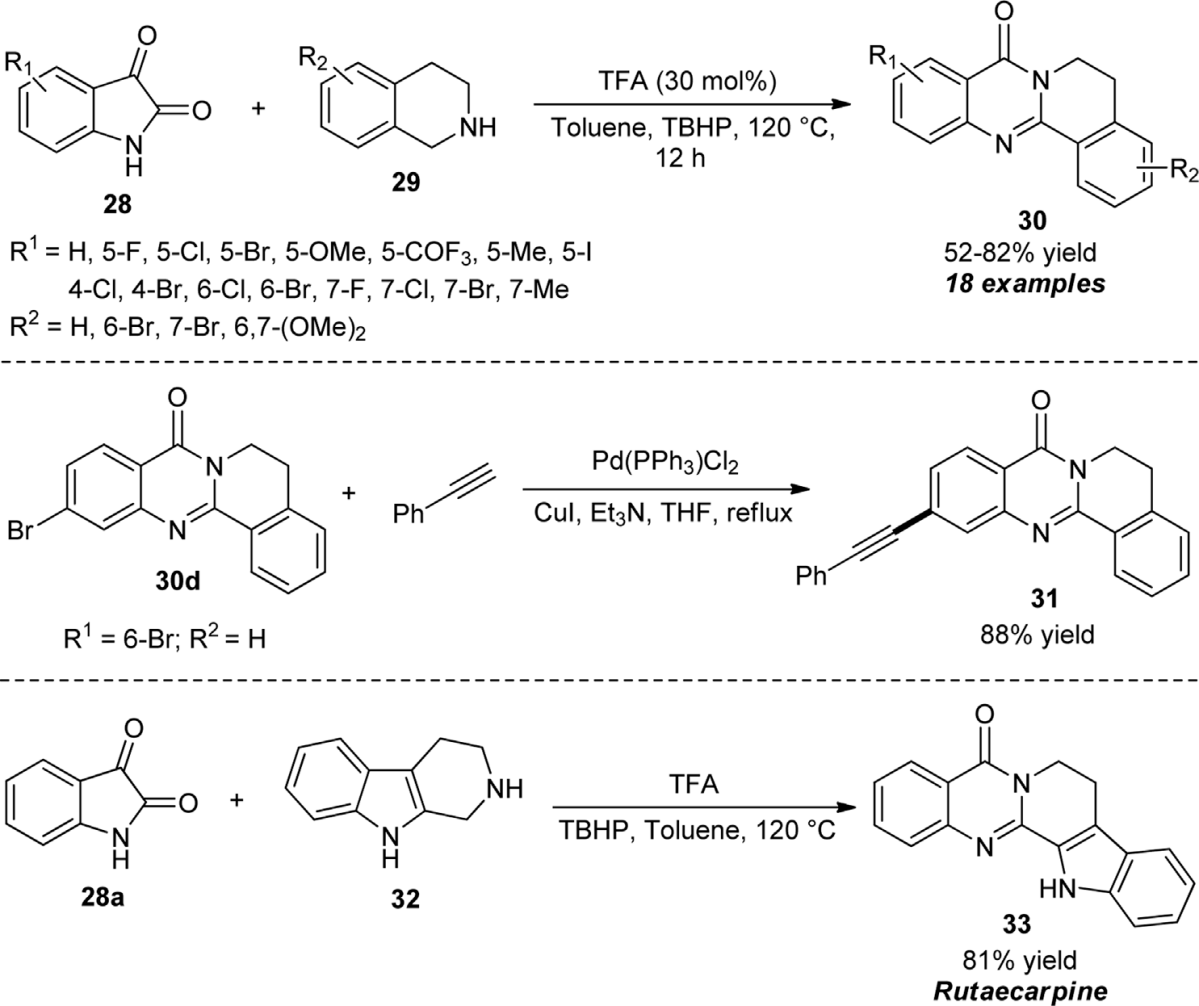
TFA/TBHP-mediated oxidative cyclization toward the synthesis of quinazolinones.

Jia et al. disclosed an acid-catalyzed oxidative cyclization strategy for the rapid construction of diverse tetracyclic quinazolinones. By using 30 mol% of trifluoroacetic acid (TFA) as the catalyst and *tert*-butyl hydroperoxide (TBHP) as the oxidant, the reaction of isatins **28** and 1,2,3,4-tetrahydroisoquinolines **29** in toluene at 120°C afforded the representative quinazolinones **30** in 52–82% yields (Scheme 8) (Jia et al., 2020). Isatins having either electron-withdrawing or donating groups on the aromatic ring smoothly participated in this reaction with no significant effect on the reaction rates. Similarly, 1,2,3,4-tetrahydroisoquinolines possessing electron-rich groups such as methoxy and halogenated groups such as bromo were established to be very efficient for this reaction. However, the reaction with alkyl-substituted benzyl amines and alkyl cyclic amines such as piperidine and pyrrolidine failed to work in this condition which marks the limitations of this approach. The synthetic application of the protocol was established by the Pd-catalyzed cross-coupling reaction of the product **30d** to furnish the product **31**. Moreover, the authors synthesized the natural alkaloid rutaecarpine **33** from the one-step reaction of isatin **28a** and 2,3,4,9-tetrahydro-1*H*-pyrido [3,4-*b*]indole **32** under optimal conditions that also contribute the practical effectiveness of the present protocol.

**SCHEME 9:**
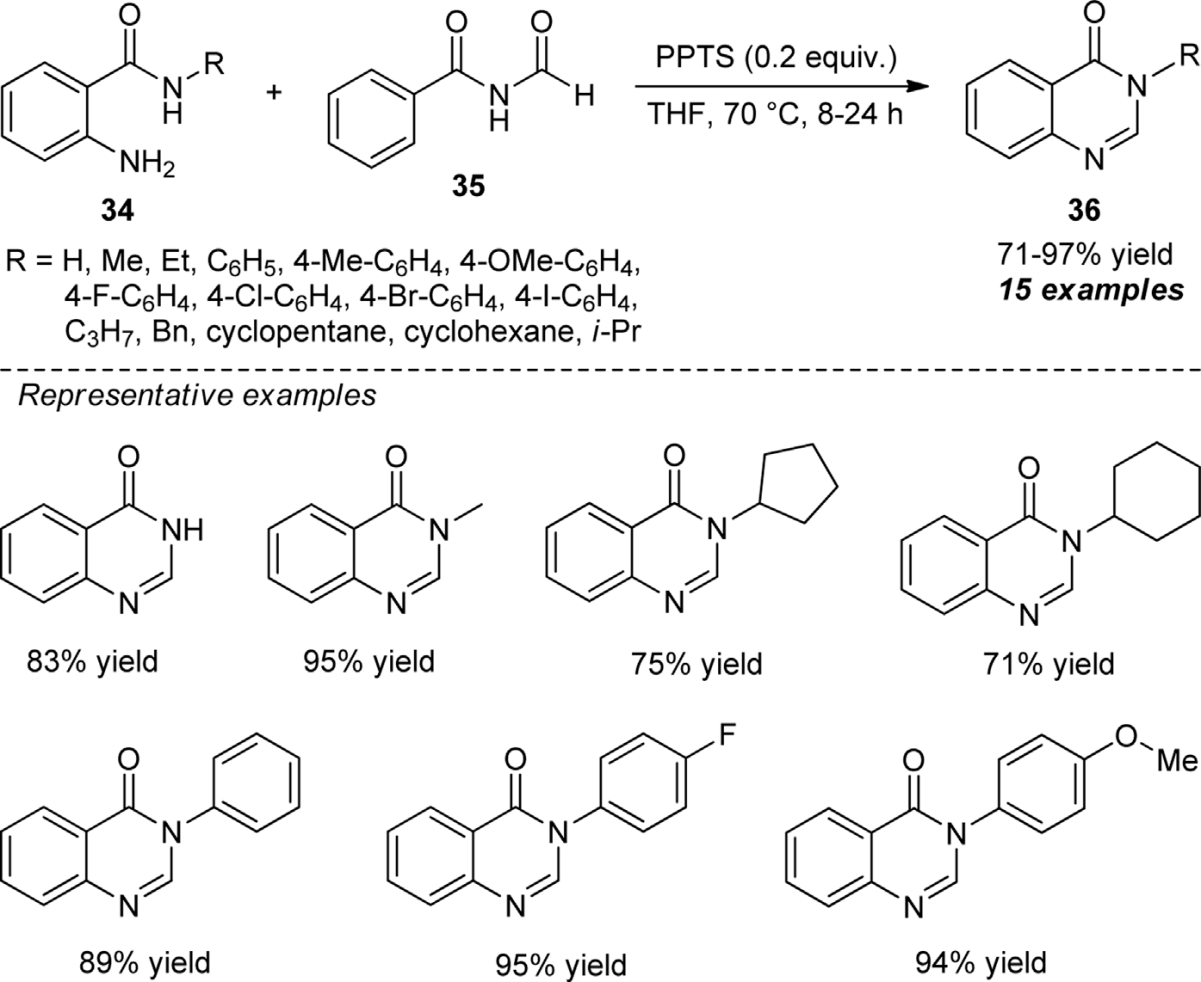
*N*-formyl imide as a carbon source to access quinazolinones by acid catalysis.

The exploitation of *N*-formyl imide as a carbon source for the metal-free synthesis of quinazolinones was developed by Huang et al. (Scheme 9) (Huang et al., 2020). With the aid of 0.2 equivalents of pyridinium *p*-toluene sulfonate (PPTS) as the acid catalyst, the treatments of diverse *N*-substituted 2-aminobenzamide **34** and *N*-formyl imide **35** using tetrahydrofuran (THF) as a solvent at 70°C, the representative products **36** were accomplished in 71–97% yields within 8–24 h. The reaction conditions tolerate a wide variety of *N*-aryl and *N*-aliphatic substitutions on the 2-aminobenzamide ring. Although the present approach offered a lot of advantages such as mild reactions, easy setup procedure, metal-free, oxidant-free, and good functional group tolerances, the reaction required a prolonged time to complete, which marks the limitations of this approach’s otherwise outstanding developments.

### Base-catalyzed two-component synthesis of quinazolinones

**SCHEME 10:**
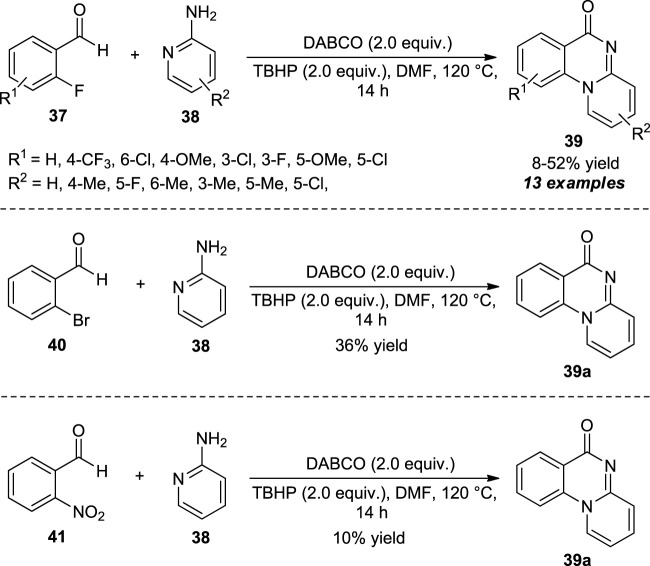
DABCO catalyzed oxidative cyclization to access quinazolinones.

Feng and Wu developed a metal-free procedure for the oxidative cyclization of 2-fluorobenzaldehyde **37** and 2-aminopyridines **38** to access quinazolinones. With the help of 1,4-diazabicyclo [2.2.2]octane (DABCO) as the basic organocatalyst and *tert*-butyl hydroperoxide (TBHP) as the oxidant, the representative products **39** were obtained in moderate to good yields (Scheme 10) (Feng and Wu, 2017). The presence of different substitutions such as methyl, chloro, and fluoro on the different positions of 2-aminopyridines leads to the corresponding products in sufficient yields, while bromo, cyano, and nitro-substituted 2-aminopyridines were unable to react under this standard condition, and only trace amounts of products were observed. This is most likely due to the reduced electron density on NH–Ar anion, thereby being unable to undergo nucleophilic substitution with electrophile **37**. On the other hand, 6-methyl substituted 2-aminopyridines provided the simple non-cyclized amide as the final product rather than the quinazolinone product due to the steric hindrance. The reaction was further investigated for other benzaldehydes, including 2-bromobenzaldehyde and 2-nitrobenzaldehyde with 2-aminopyridines under optimal conditions, and the respective quinazolinone products were found to be formed in 36 and 10% yield, respectively.

**SCHEME 11:**
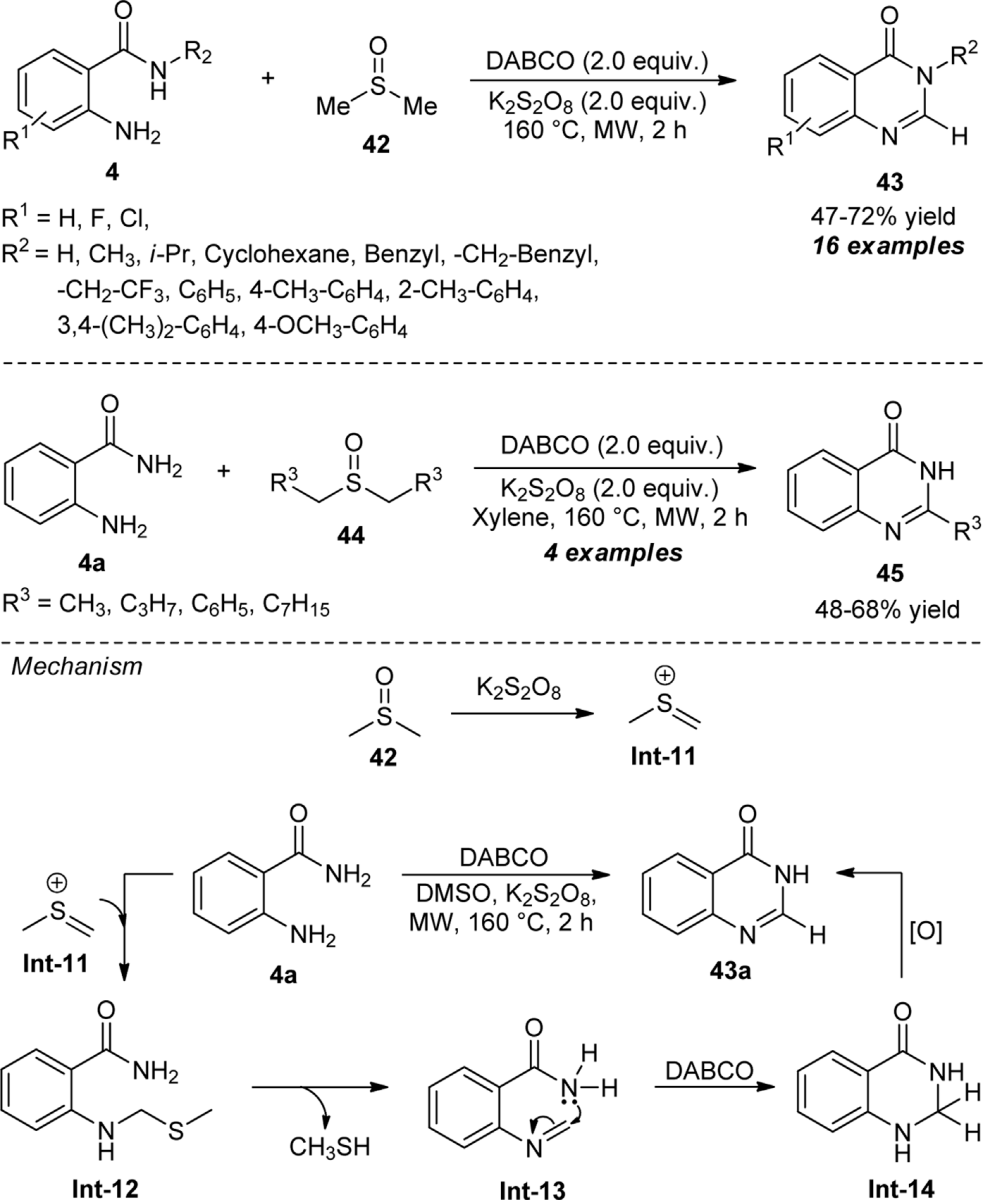
DABCO catalyzed construction of quinazolinones using DMSO as the methine source.

Lee et al. demonstrated the exploitation of dimethyl sulfoxide (DMSO) as the carbon source for the assembly of quinazolinones under transition-metal-free conditions. With the aid of 1,4-diazabicyclo [2.2.2]octane (DABCO) as the basic catalyst and potassium persulfate (K_2_S_2_O_8_) as the additive, the microwave-assisted reaction of 2-amino benzamides **4** with DMSO furnished the representative products **43** in 47–72% yields within 2 h (Scheme 11) (Lee et al., 2019). The reaction conditions were well-tolerated for both *N*-unsubstituted and *N*-substituted 2-amino benzamides, with a slight change in the yield of the products. With *N*-substituted amides, all aliphatic and aromatic groups having electron-releasing substituents react smoothly to afford the products in quantitative yields. This is due to an increase in the nucleophilicity of the amide, thereby increasing the yield of the products by favoring annulation, whereas trifluoromethyl substituted amide also provided the product efficiently, albeit with a 47% yield. The strong electron-withdrawing groups such as cyano and nitro on the *N*-aryl ring of **4** decomposed under this reaction condition which marks the limitations of this approach. In addition to these, a wide variety of dialkyl-substituted sulfoxides was established to be very effective for this reaction to deliver the quinazolinone products **45**. The proposed mechanism involves the reaction of DMSO with K_2_S_2_O_8_ to *in situ* form the sulfenium ion **Int-11** which condensed with **4a** to form **Int-12**. The intermediate **Int-13** was formed from **Int-12** after the elimination of CH_3_SH, which can then undergo intramolecular annulation to produce **Int-14**. The final autooxidation of **Int-14** yields the final product **43a**.

**SCHEME 12:**
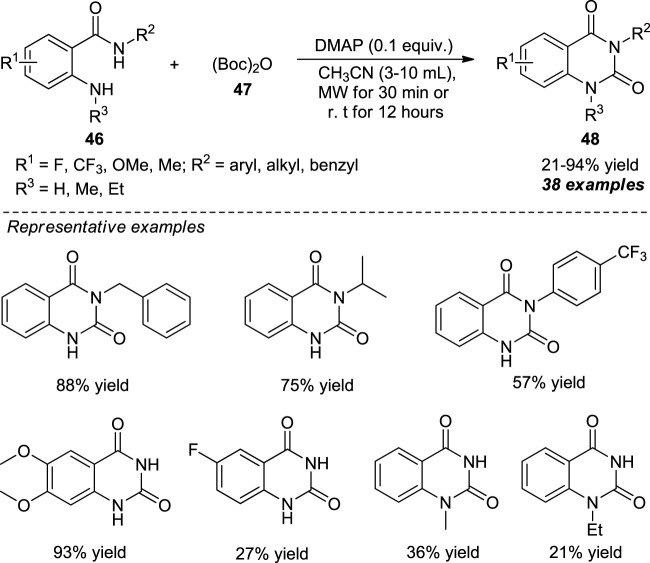
DMAP catalyzed one-pot synthesis of quinazolinones.

Chen et al. devised a microwave-assisted metal-free one-pot strategy for the synthesis of quinazolin-2,4-diones **48** from 2-aminobenzamides **46** and di-*tert*-butyl decarbonate (Boc)_2_O **47** as the precursor which constructed the carbonyl moiety at the 2-position of quinazolinediones. With the aid of 4-dimethylaminopyridine (DMAP) as the base catalyst, the representative product **48** could be accomplished either by microwave irradiation for 30 min or simple stirring at room temperature for 12 h in poor to excellent yields (Scheme 12) (Chen et al., 2020). Diverse substitutions on the nitrogen atom of 2-aminobenzamides by alkyl, benzyl, and aryl groups smoothly participated in this reaction. When *R* ^2^ was an electron-donating group such as alkyl or benzyl, the yield of the product was increased as compared to aryl substitution with various electron-deficient groups that offered a lower yield, albeit with *p*-methoxy substitutions that yield 82% of the product. Furthermore, 2-aminobenzamides possessing methoxy or methyl group (R^1^ = Me, OMe) furnish the products with greater yields due to an increased nucleophilicity of the amino group, whereas electron-deficient groups (R^1^ = F, CF_3_) gave very low yields. Conversely, 1-substituted quinazoline-2,4-diones (R^3^ = Me, Et) have also been synthesized by this reaction but in very low yields.

**SCHEME 13:**
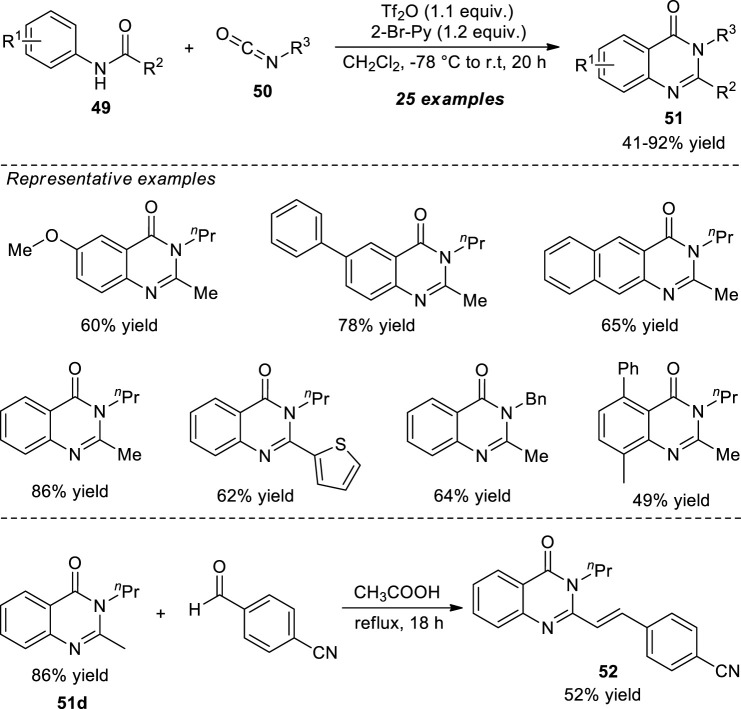
Metal-free chemoselective one-pot construction of quinazolinones *via* reaction with isocyanates.

Lin et al. demonstrated the one-pot construction of various 2,3-dialkyl substituted quinazolinones *via* the aza-annulation of secondary amides and isocyanates. With the aid of 2-bromopyridine as the base catalyst, the triflic anhydride (Tf_2_O) activated *N*-aryl amide **49** and isocyanates **50** afforded the representative quinazolinone products **51** in 41–92% yields. Broad functional group tolerances, mild setup procedure, and good chemoselectivity are some of the salient features of this protocol. The synthetic application of the present protocol was established by the acid-catalyzed reaction of product **51d** with 4-formylbenzonitrile to form the biologically active 2-styrylquinazolinones such as product **52** (Scheme 13) (Lin et al., 2021).

### Other organocatalytic two-component synthesis of quinazolinones

**SCHEME 14:**
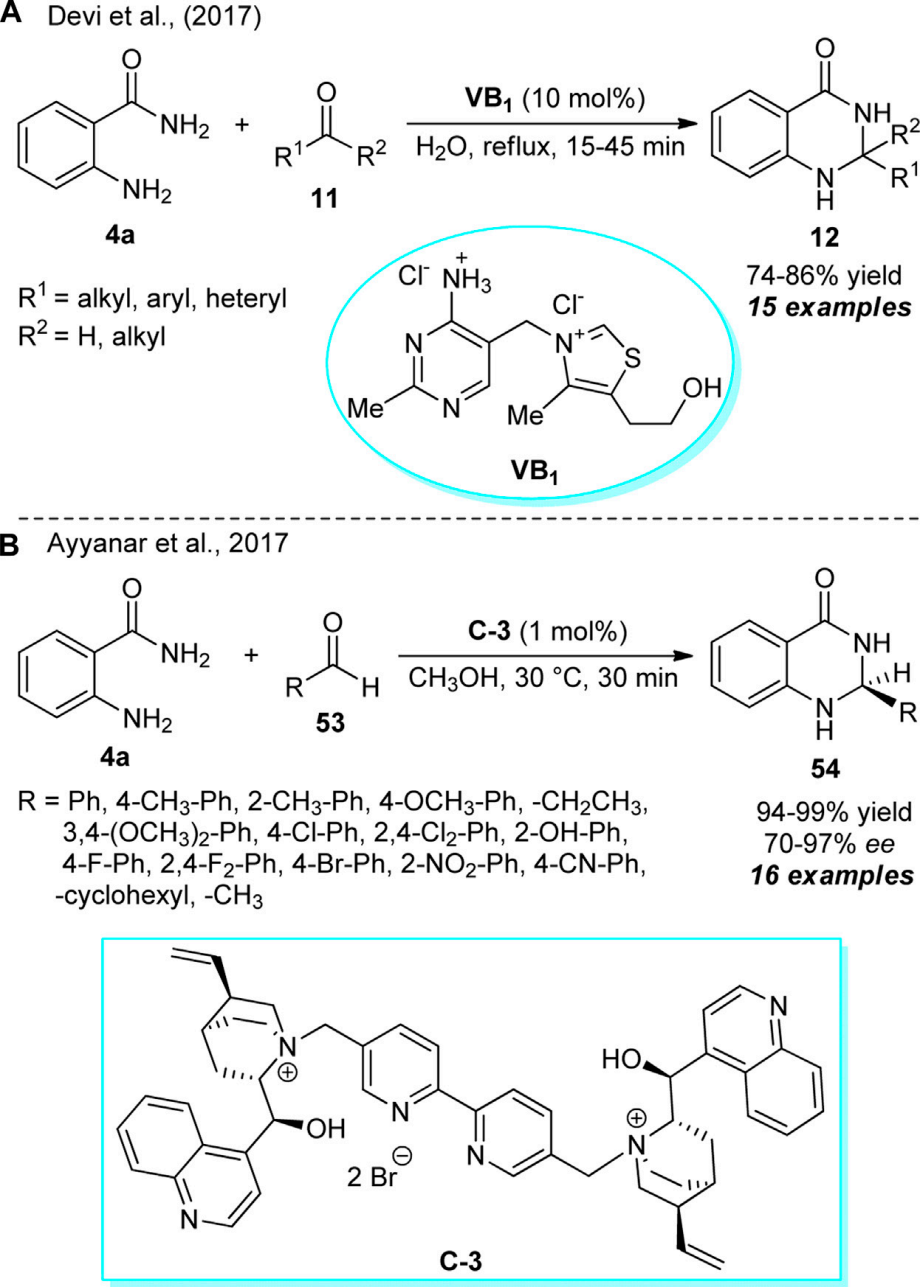
Vitamin B_1_ catalyzed water enabled synthesis of quinazolinones **12** **(A)** and enantioselective construction of quinazolinones **53** by organocatalyst **C-3** **(B)**.

The introduction of thiamine hydrochloride, commonly known as vitamin B_1_ (VB_1_), as an efficient organocatalyst for the construction of 2,3-dihydroquinazolinones was developed by Devi et al. (Scheme 14A) (Devi et al., 2017). With water as the green mother nature solvent, the treatments of 2-aminobenzamide **4a** and diverse aldehydes and ketones **11** were found to proceed smoothly under reflux conditions to deliver the representative products **12** in 74–86% yields. The reaction required only 10 mol% of the catalyst and was completed within a very short duration of time. To broaden the scopes of the reaction, a diverse range of heteroaryl and aryl-substituted aldehydes with varying electron-donating and electron-deficient substituents were explored and recognized to be very compatible with this reaction. On the other hand, ketones such as cyclopentanone, cycloheptanone, and acetone were well-tolerated, whereas acetophenone failed to deliver any product. The utilization of water as the solvent, reusable catalytic system, and low cost of the catalyst makes this protocol environmentally benign.

Around the same time, a highly convergent and facile catalytic enantioselective construction of 2,3-dihydroquinazolinones was developed by Ayyanar et al. (Scheme 14B) (Ayyanar et al., 2017). With the support of 1 mol% of chiral organocatalyst **C-3**, the reaction of 2-aminobenzamide **4a** and aldehydes **53** in methanol at 30 °C afforded the representative optically active products **54** in 94–99% yields with moderate to excellent enantioselectivity. The reaction conditions were very suitable for a vast array of aryl aldehydes to work efficiently with this reaction, and for ortho, para, or meta substitutions, better stereo control was achieved. In addition to demonstrating cyclohexanone as the reactive partner for this reaction, the reaction was not completed with aliphatic aldehydes (R = Me, C_3_H_7_). Interestingly, this reaction does not require any co-catalyst or additives to improve the enantioselectivity of the respective products.

## Synthesis of quinazolinones from multi-component organocatalytic reactions

### Acid-catalyzed multi-component synthesis of quinazolinones

Recently, multi-component reactions (MCRs), which allow the formation of multiple bonds in a single operation, have been demonstrated as a promising tool for the creation of diverse molecular structures with enhanced efficiency, reduced waste, and high atom economy from easily accessible simple and inexpensive starting materials by effortless mixing of the reactant (Rotstein et al., 2014; Borah et al., 2022c). The ability to accomplish the requisite products in “one-pot” by operationally simple workup procedure without using complex purification techniques and avoiding the isolation of the reaction intermediate features multi-component reaction, a powerful strategy for green or sustainable synthesis (Borah et al., 2022b).

**SCHEME 15:**
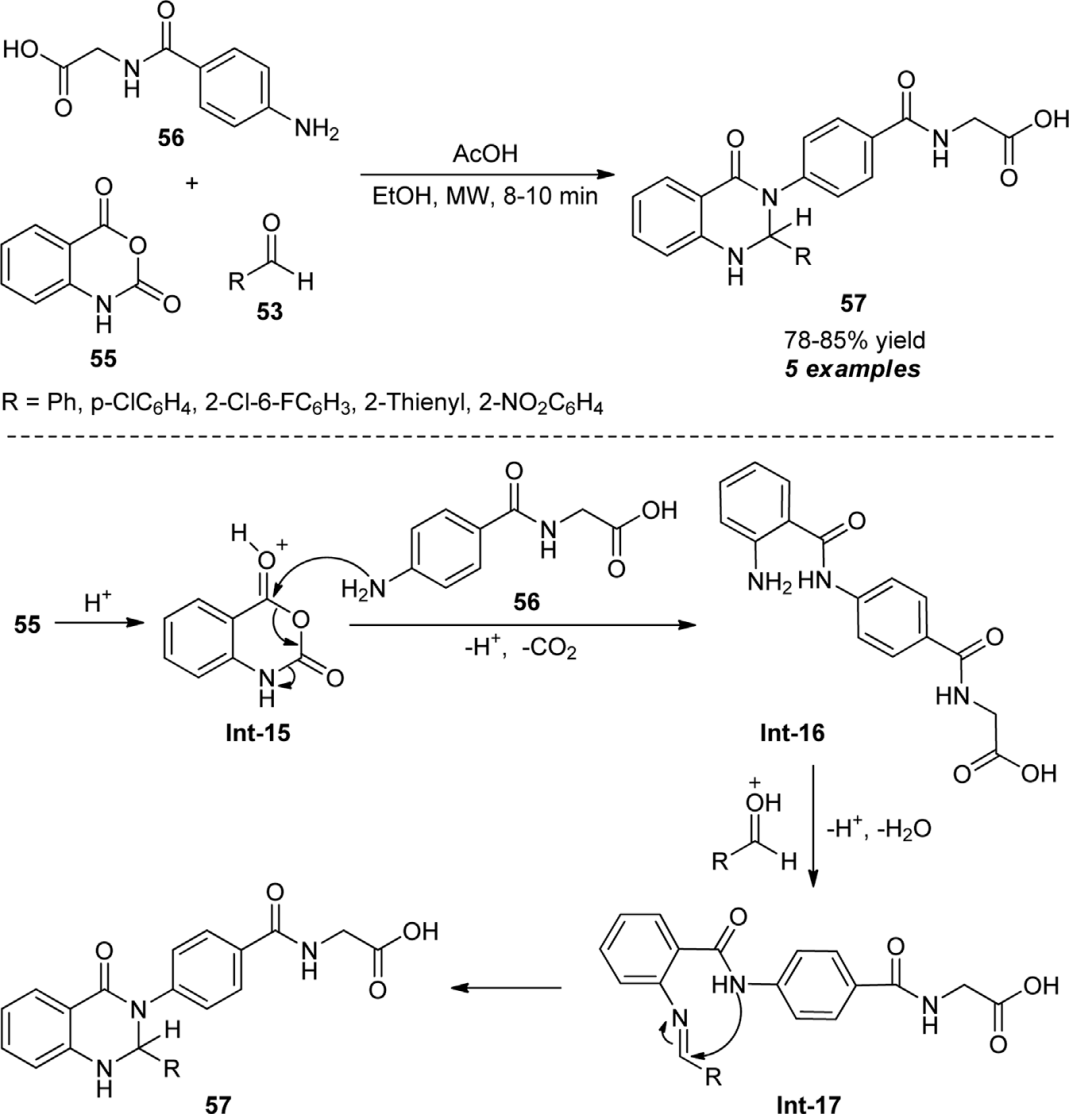
Acetic acid-catalyzed microwave-assisted synthesis of 2,3-dihydroquinazolinones.

Recognizing these features, Fozooni and Firoozi devised a one-pot procedure for the synthesis of 2,3-dihydroquinazolinones by introducing a microwave-assisted three-component reaction of isatoic anhydride **55**, aldehydes **53**, and 2-(4-aminobenzamido)acetic acid **56** (Scheme 15) (Fozooni and Firoozi, 2015). With the aid of acetic acid (AcOH) in ethanol, the representative products **57** were accomplished in 78–85% yields within 8–10 min. Aryl and heteroaryl substituted aldehydes have been selected to be very compatible for this reaction. Although the reaction proceeded under both microwave irradiation and the conventional method, the low yield of the products was observed by the conventional method and required a longer reaction time as compared to microwave techniques. The exploitation of microwaves not only enhances the yield but also reduces the reaction times and provides a clean pathway toward the products. Notwithstanding these developments, the narrow substrate scopes constitute the limitation of this protocol and call for further developments. The overall process proceeded through the decarboxylative reaction between acid-activated isatoic anhydride **Int-15** and amine **56** to form an intermediate **Int-16** which on reaction with activated aldehydes produce the imine intermediate **Int-17**. The subsequent cyclization of **Int-17** delivered the products **57**.

**SCHEME 16:**
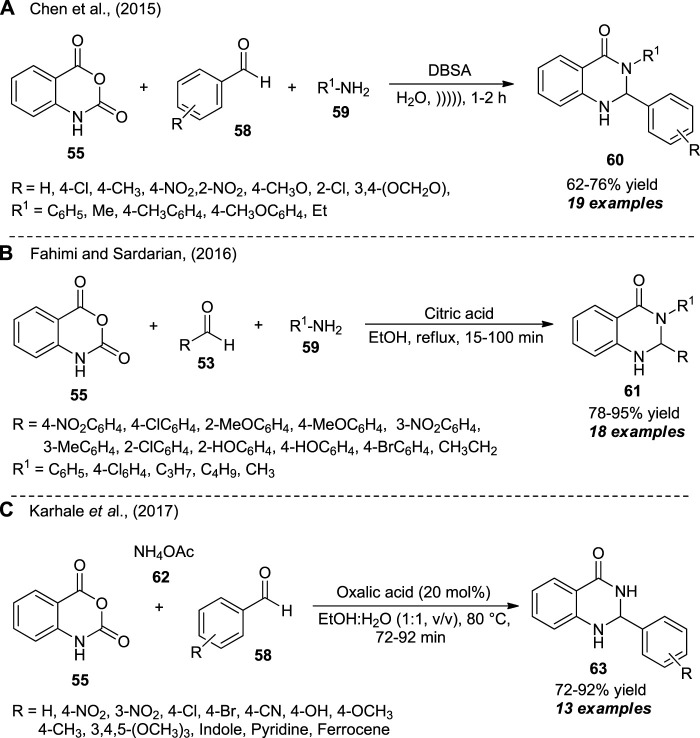
Domino synthesis of quinazolinones in different reaction conditions as developed by Chen et al., **(A)**, Fahimi **(B)** and Karhale et al., **(C)**.

The introduction of ultrasound irradiation as a highly efficient and environmentally benign activation method for the one-pot assembly of 2,3-dihydroquinazolinones was realized by Chen et al. (Scheme 16A) (Chen et al., 2015). With water as the green solvent system and dodecylbenzene sulfonic acid (DBSA) as the catalyst, the ultrasound-assisted three-component reaction of isatoic anhydride **55**, aldehydes **58**, and aniline **59** afforded the representative quinazolinone products **60** in 62–76% yields within 1–2 h. In a similar manner, Fahimi and Sardarian synthesized a series of 2,3-substituted quinazolinones **61** from the reaction of isatoic anhydride **55**, aldehydes **53**, and aniline **59** by using citric acid as the catalyst in ethanol under reflux conditions. A broad variety of electron-deficient or electron-donating substituents have been smoothly worked under the standard conditions, and a total of 18 compounds were isolated in 78–95% yield (Scheme 16B) (Fahimi and Sardarian, 2016). Later, Karhale et al. disclosed the oxalic acid-catalyzed three-component reaction of isatoic anhydride **55**, aldehydes **58**, and ammonium acetate **62** for the rapid access to quinazolinone derivatives **63** in 72–92% yields (Scheme 16C) (Karhale et al., 2017). The broad functional groups, mild setup procedures, short reaction times, easy isolation, column-free, waste-free, and metal-free were some of the advantages of all the three procedures developed. However, the yield of the products in all the cases needs to be improved, which leaves a gap for further developments.

**SCHEME 17:**
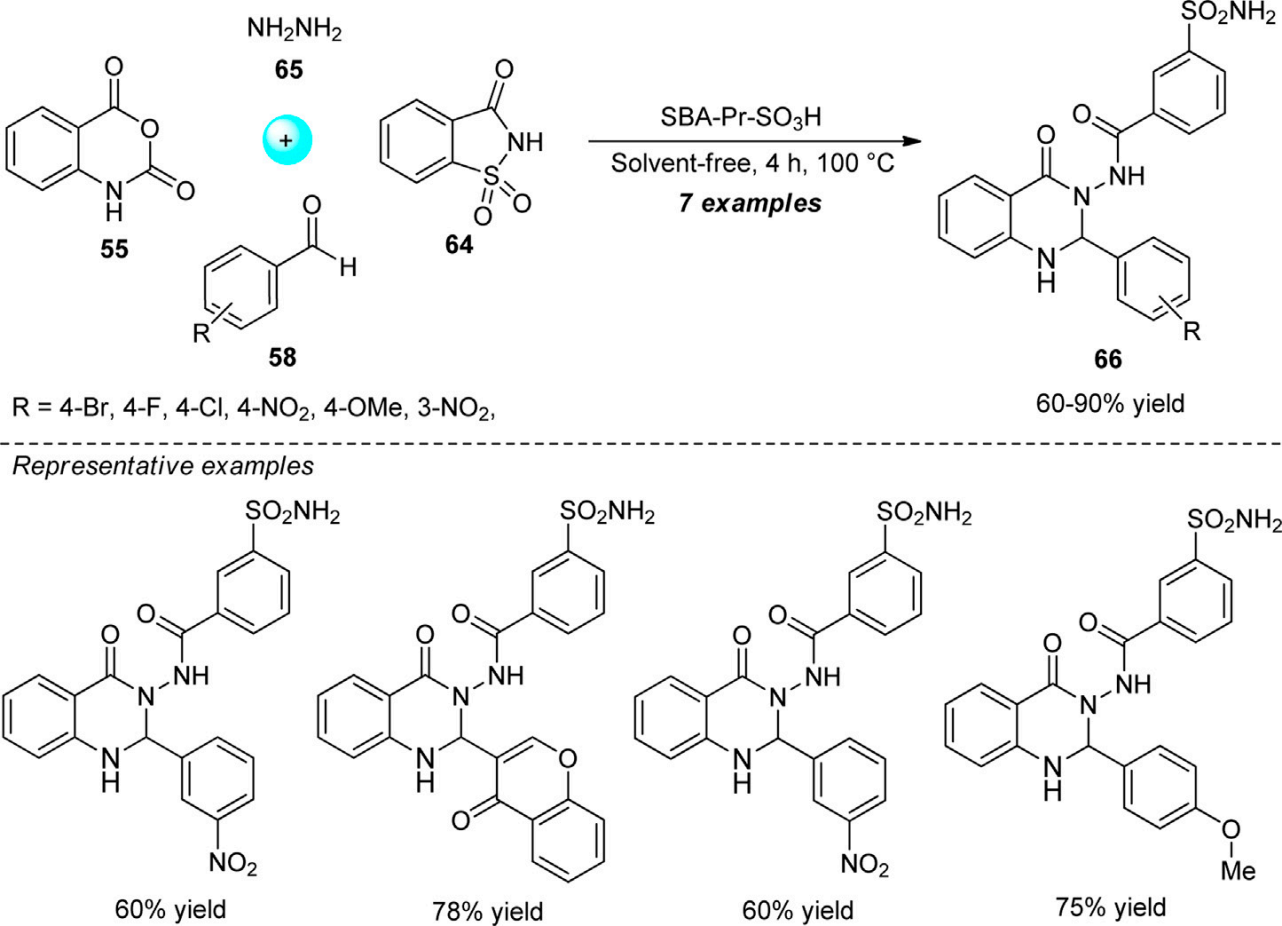
Solvent-free one-pot consecutive synthesis of quinazolinone sulfonamides.

A highly convenient one-pot strategy for the consecutive synthesis of quinazolinone sulfonamides was designed by Balalaie et al. from the four-component reaction of isatoic anhydride **55**, aldehydes **58**, saccharin **64**, and hydrazine hydrate **65** (Scheme 17) (Balalaie et al., 2017). With the aid of propyl sulfonic acid functionalized SBA-15 (SBA-Pr-SO_3_H) as a heterogeneous catalyst in solvent-free conditions, the representative products **66** have been isolated in 60–90% yields after 4 h. The avoidance of solvents, utilization of readily accessible starting materials, and easy work-up procedure, are key salient features of this protocol. However, the narrow substrate scopes mark the limitations of this strategy.

**SCHEME 18:**
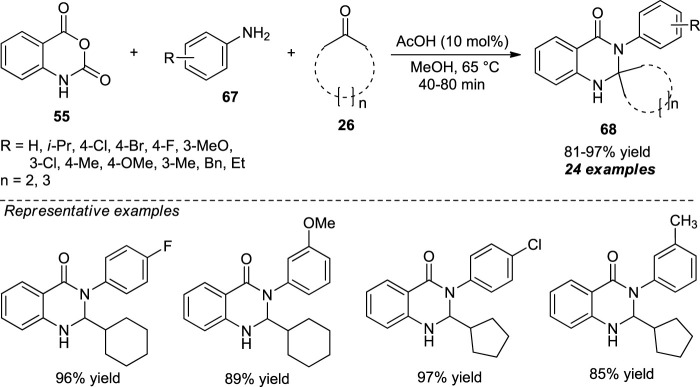
Acetic acid-catalyzed one-pot construction of quinazolinones.

A concise organocatalytic one-pot approach for the facile access to spiro-fused quinazolinones by means of acetic acid catalyzed three-component reaction of isatoic anhydride **55**, aryl amines **67**, and cyclic ketones **26** was demonstrated by Ramesh et al. With the aid of 10 mol% of the catalyst, the desired products **68** have been obtained in 81–97% yields (Scheme 18) (Ramesh et al., 2018). The reaction condition tolerates a broad range of functional groups with a slight decrease in yield in the case of aliphatic amines. This is presumably due to their high nucleophilicity. Overall, it has the advantages of mild reaction conditions, short reaction time, experimental simplicity, and excellent yields.

**SCHEME 19:**
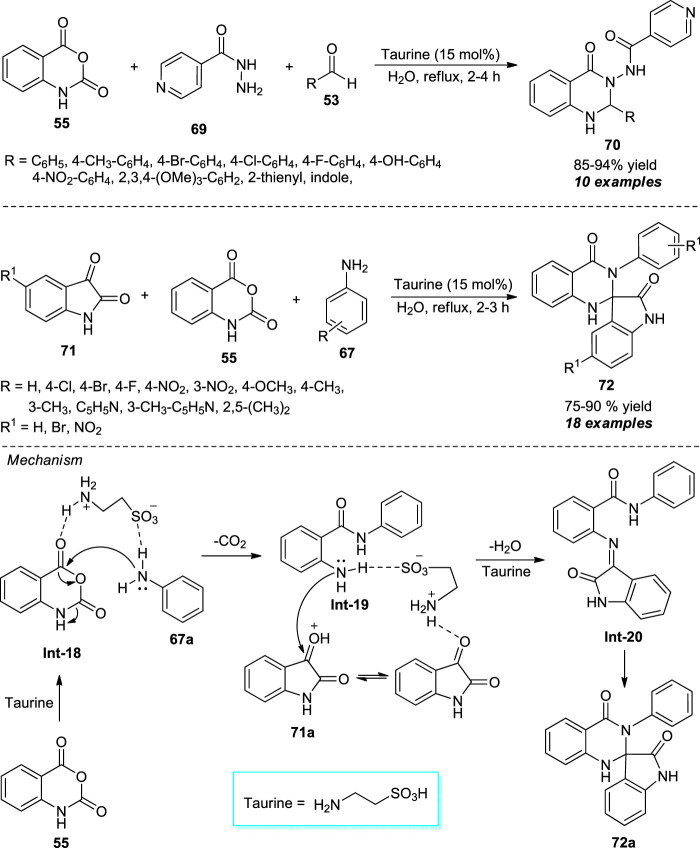
Taurine catalyzed one-pot construction of quinazolinones in water.

Taking advantage of the bifunctional donor-acceptor reactivity of 2-aminoethanesulfonic acid (taurine), Chate et al. disclosed a facile and efficient one-pot procedure for the assembly of diverse quinazolinone derivatives **70** under mild conditions (Scheme 19) (Chate et al., 2020). With the aid of 15 mol% of taurine as the organocatalyst, the three-component reaction of isatoic anhydride **55**, isoniazid **69**, and aldehydes **53** using water as the green solvent system, the corresponding products **70** have been obtained in 85–94% yields within 2–4 h under reflux conditions. Inspired by this result, they further investigated the reaction between isatins **71**, isatoic anhydride **55**, and amines **67** under standard conditions. Interestingly, the reaction afforded the spiro-oxindole-bearing quinazolinone products 75–90% yields **72** in. A wide variety of aryl and heteroaryl substituted aldehydes and amines were well-tolerable for both reactions. The practical application of the protocol was established by demonstrating the recyclability experiments of the catalyst up to three consecutive cycles with a slight change in catalytic properties. The formation of product **72a** can be explained by the initial nucleophilic addition of amine **67a** to taurine-activated isatoic anhydride followed by decarboxylation to form the intermediate **Int-19**. This intermediate on condensation with protonated isatin **71a** delivered imine intermediate **Int-20**, and the subsequent intramolecular cyclization of **Int-20** yields the final product **72a**.

**SCHEME 20:**
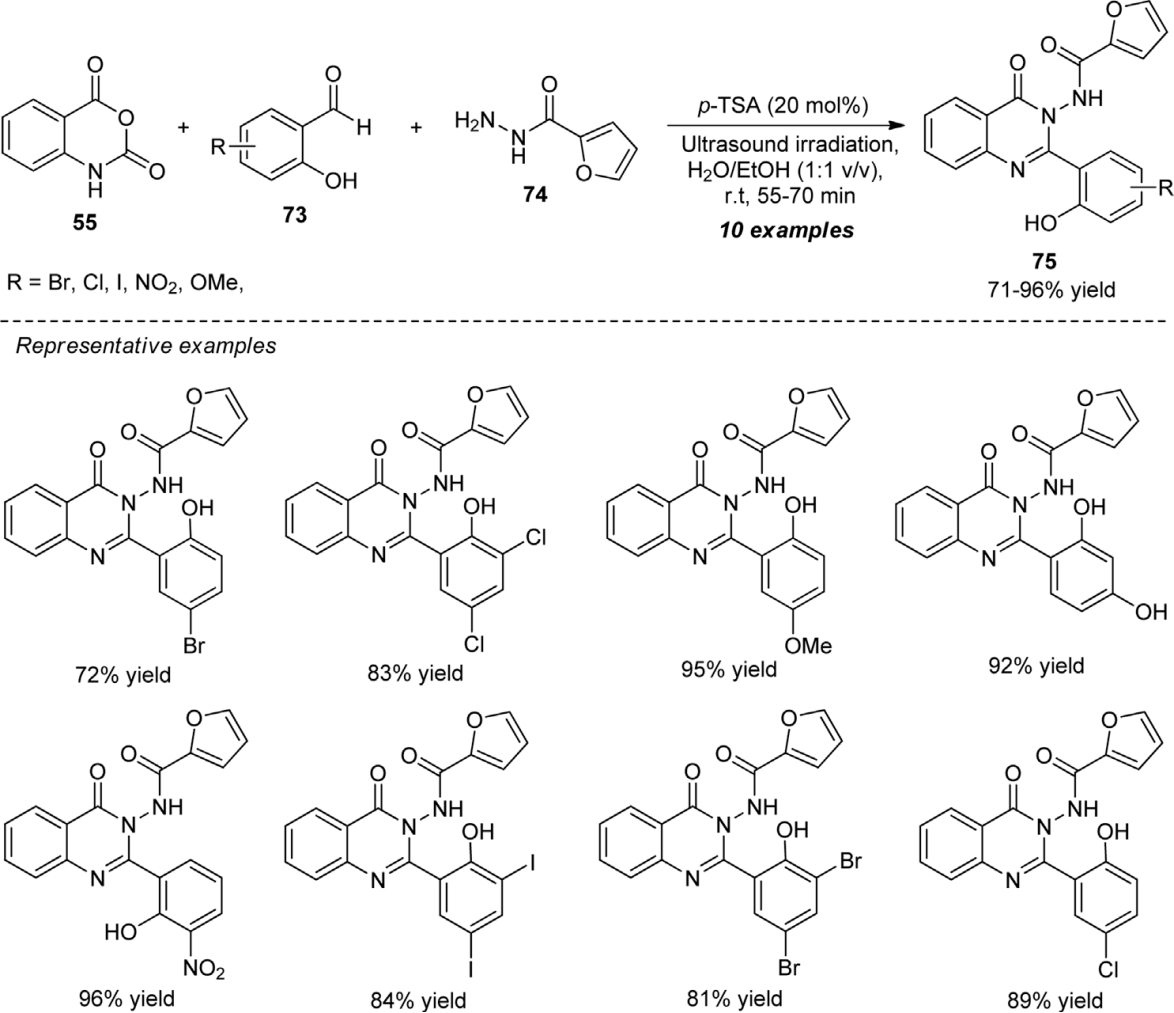
Acid-catalyzed multi-component synthesis of quinazolinones under sonication.

Dige et al. explored a highly convenient ultrasound-assisted strategy for the Brønsted acid-catalyzed construction of a variety of quinazolinones by a one-pot multi-component reaction of isatoic anhydride **55**, 2-furoic hydrazides **74**, and various substituted salicylaldehydes **73** (Scheme 20) (Dige et al., 2019). By employing 20 mol% of *p*-toluene sulfonic acid (*p*-TSA) in an aqueous ethanolic solution, the representative quinazolinone products **75** have been accomplished in 71–96% yields within 55–70 min of ultrasonication at room temperature. Pleasingly, it was found that salicylaldehydes possessing different electron-rich and electron-poor groups worked suitably in this reaction without having an adverse effect on the product yield. A few of the synthesized compounds were also shown to be tyrosinase enzyme inhibitors. Some of the main advantages of this strategy include the use of sound waves as an effective green way, combined green solvents, quick reaction times, and simple extraction methods.

### Base-catalyzed multi-component synthesis of quinazolinones

**SCHEME 21:**
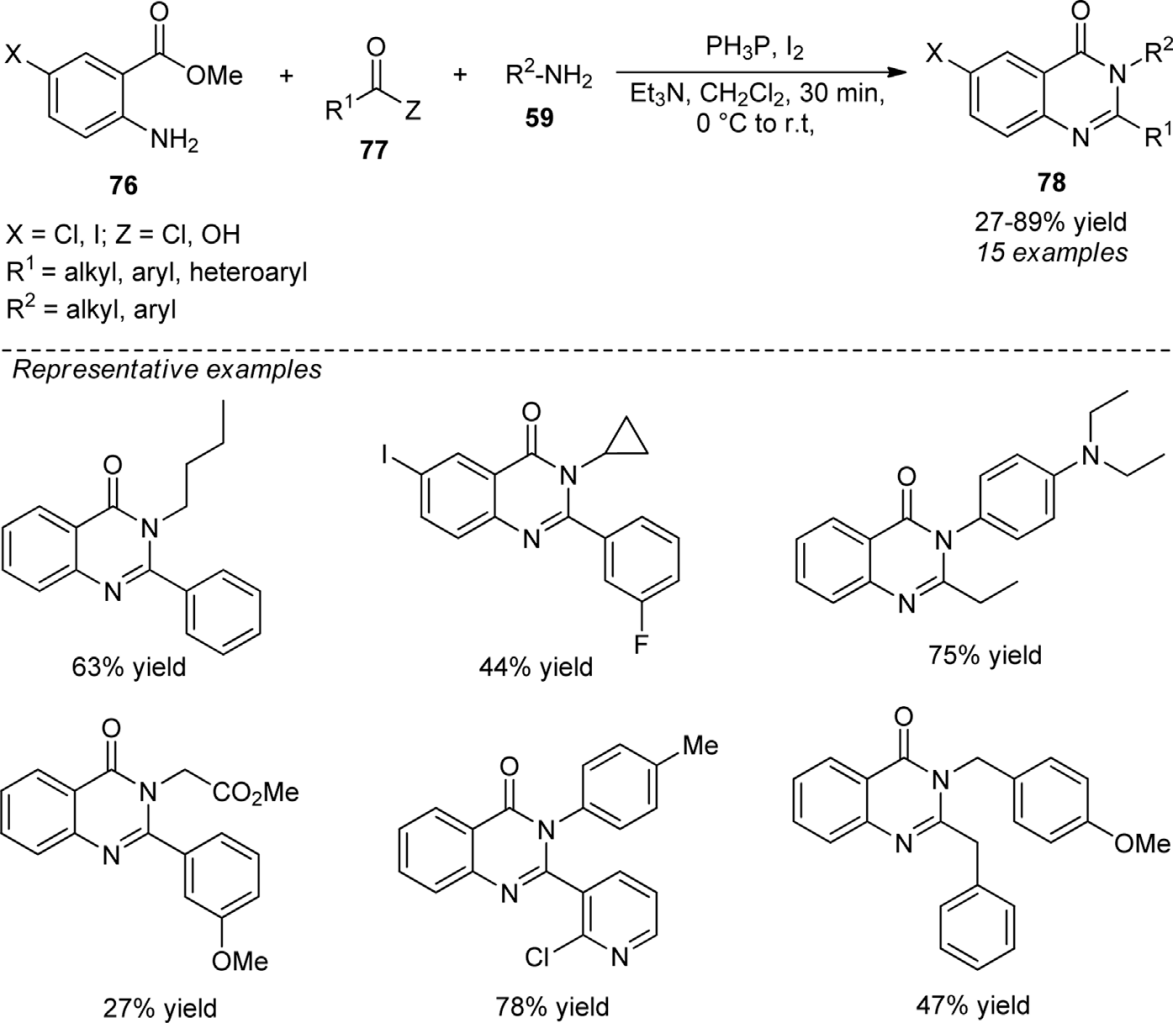
Ph_3_P−I_2_ mediated one-pot construction of quinazolinones.

Phakodee et al. disclosed a highly efficient Ph_3_P−I_2_ promoted synthesis of quinazolinones **78** from the treatment of methyl anthranilate **76**, carboxylic acid or acid chloride **77**, and amine **59**. Diverse substitutions on ring **76** and various amines were found to be tolerable to deliver the products in 27–89% yield. Although the synthesis of quinazolinones from acid chlorides has smoothly proceeded, the synthesis directly from carboxylic acids was found to be very difficult and provides a low yield of the products. This might be due to the difficulties in the amide bond formation from aliphatic acids. Broad functional group tolerance, mild reaction conditions, and readily accessible starting materials are some of the advantages of this protocol (Scheme 21) (Phakhodee et al., 2017).

**SCHEME 22:**
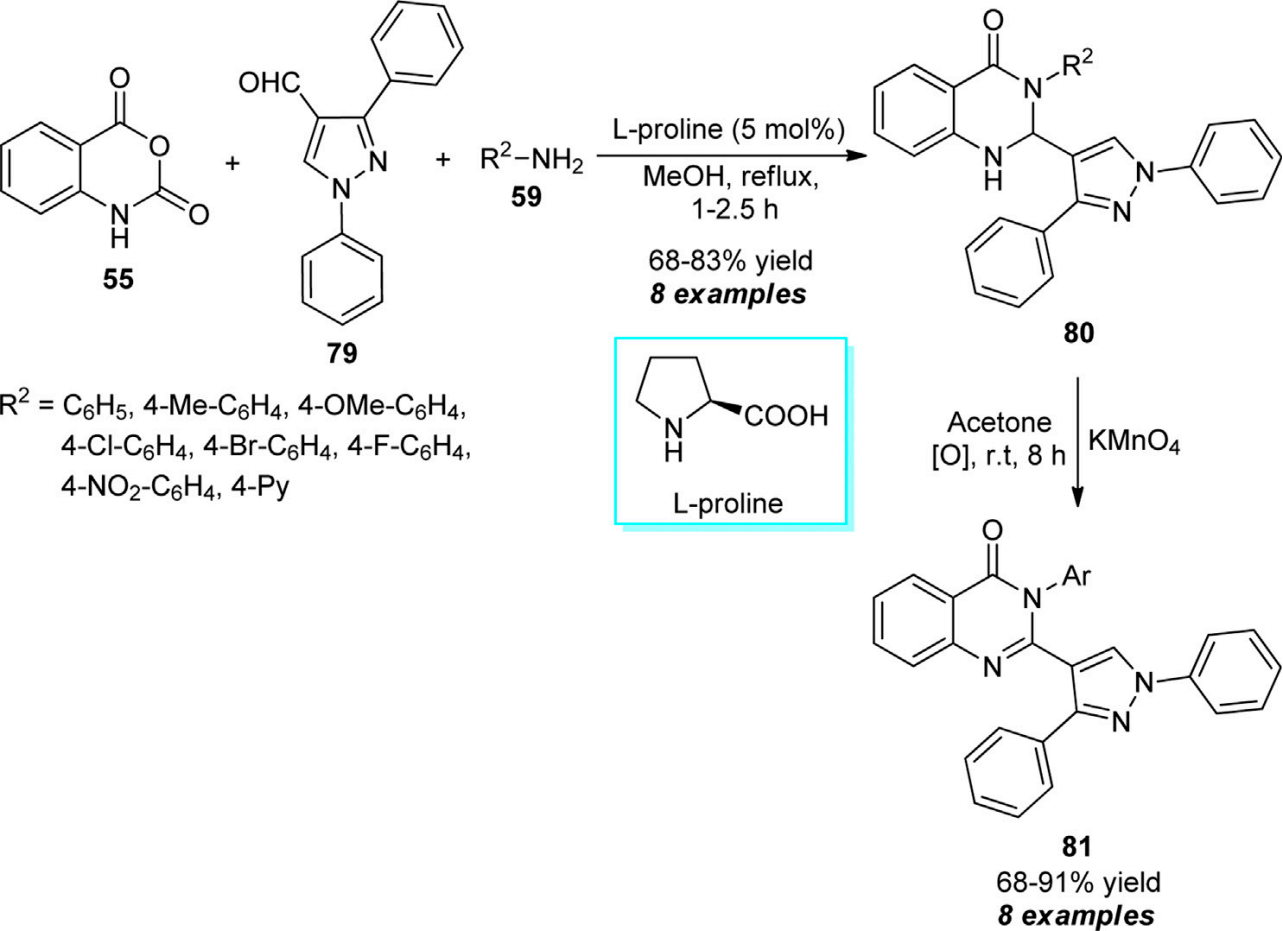
l-proline catalyzed construction of quinazolinones by using KMnO4 as an oxidating agent.

Treatment of isatoic anhydride **55**, pyrazole substituted aldehydes **79**, and diverse amines **59** proceeded smoothly in the presence of 5 mol% of l-proline to furnish the desired dihydro-quinazolinone products **80** in moderate to good yields, which on oxidation with potassium permanganate (KMnO_4_) yielded the quinazolinones bearing pyrazole core **81** after 8 h in 68–91% yields (Scheme 22) (Mehta et al., 2014). Most of the synthesized compounds have been established as antimicrobial, antifungal, and antituberculosis agents. The operational simplicity, green solvent system, mild reaction conditions, and biologically active compounds are some key features of this approach. However, the narrow substrate scopes with the low yield of the products point toward the drawback of this procedure.

### Other organocatalytic multi-component synthesis of quinazolinones

**SCHEME 23:**
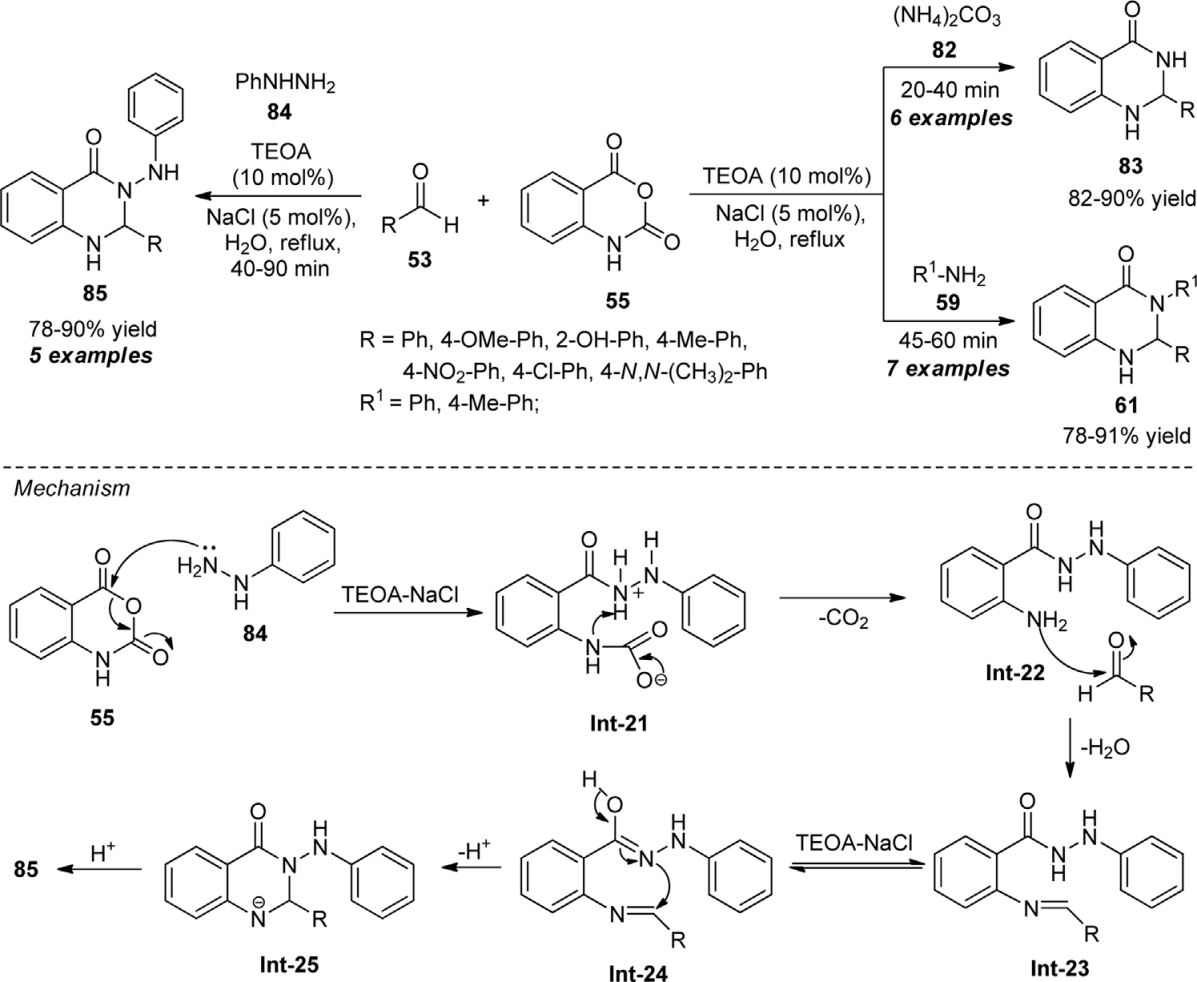
TEOA in combination with NaCl-catalyzed synthesis of quinazolinones in an aqueous medium.

A facile and eco-friendly domino multi-component strategy for the synthesis of quinazolinone derivatives was developed by Khandebharad et al., using triethanolamine (TEOA) as the catalyst (Scheme 23) (Khandebharad et al., 2020). With the aid of 10 mol% of the catalyst and 5 mol% of NaCl in the aqueous medium, the one-pot reaction of isatoic anhydride **55**, aldehydes **53** with various nitrogen sources such as ammonium carbonate **82**, amine **59**, or phenyl hydrazine **84** under reflux conditions delivered the representative products **61**, **83**, and **85** in moderate to good yields respectively. The utilization of sodium chloride (NaCl) salt in this process increases the hydrophobic effect in aqueous media and controls the formation of micelles, thereby forming the desired product significantly in a cleaner way. On the other hand, because the reaction is temperature-dependent, a drop in temperature favors the development of micellar structures by reducing the hydration of the hydrophilic head group. Consequently, as the temperature rises, the micellization process occurs at a lower concentration. This process has several key characteristics that make it an effective and promising synthetic approach for the synthesis of quinazolinone derivatives, including straightforward reaction conditions, outstanding yield, a wide substrate range, and an easy work-up procedure.

**SCHEME 24:**
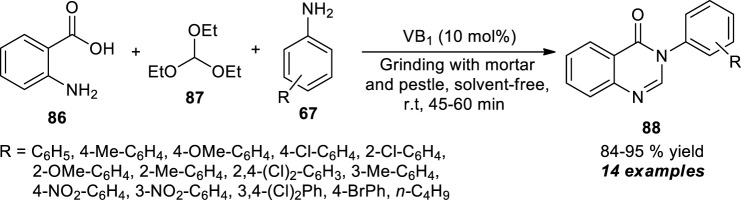
Mechanochemical one-pot synthesis of quinazolinones catalyzed by thiamine hydrochloride.

Kawade et al. developed a facile mechanochemical multi-component strategy for the efficient access to quinazolinone derivatives by the one-pot treatments of anthranilic acid **86**, triethyl orthoformate **87**, and aromatic amines **67** (Scheme 24) (Kawade et al., 2016). Grinding the reactants with the help of a mortar and pestle by using 10 mol% of thiamine hydrochloride (VB_1_) as the organocatalyst, the representative quinazolinone products **88** have been accomplished in 84–95% yields within 45–60 min. The reaction is not vulnerable to electron-donating and withdrawing groups on aromatic amine, not showing any considerable difference in the yield of the final products. In contrast, the reaction did not proceed with aliphatic amines, which marks the shortcoming of the current approach.

## Conclusion and future perspectives

Recognizing the widespread distribution of quinazolinone heterocycle in the key fragments of natural alkaloids and pharmacologically active molecules, the elegant synthesis of these heterocycles has always received tremendous attention over the last decades to the present. However, in this 21st century, to move toward more green and sustainable chemistry, the development of new synthetic pathways by exploring the potentiality of simple easily accessible raw materials to construct highly functionalized quinazolinone molecules that will hold huge pharmaceutical applications is extremely demanding but challenging.

Notwithstanding the tremendous progress accomplished in the transition-metal-catalyzed construction of quinazolinone, most of them require other non-commercial supporting ligands, co-catalyst(s), additives, high energy conditions, and long reaction times. On the other hand, the high cost involved in the preparation of metal catalyst(s) marks the major limitations in quinazolinone chemistry. In addition, they are very sensitive to air and moisture. The removal of transition metal catalysts from a reaction mixture which is particularly crucial to the pharmaceutical industry often becomes a formidable challenge and is very expensive, which points toward the failure of green and more sustainable synthesis. Consequently, the occurrence of transition metal catalysts even at the lowest loading corresponds to disadvantageous effects on the environmentally friendly nature of the chemical process.

In this review article, we have demonstrated the recent organocatalytic approaches for the assembly of diverse quinazolinone derivatives. The ability to accomplish these heterocycles by means of transition-metal-free organocatalytic conditions with an immediate decrease in the cost of the transformations and the utilization of easily accessible starting material and green solvent system in most cases features the advantages of the developed synthetic procedures. From the aforementioned observations, it is clear to conclude that the exploitation of organocatalysts in different solvent systems such as water, ethanol, and methanol and solvent-free conditions offered the desired quinazolinones products in very good to excellent yields. Moreover, the reaction with organocatalysis does not necessitate any other supporting ligands, co-catalysts, oxidants, and other expensive materials in most cases, which demonstrates the sustainability and eco-friendly nature of the transformation. In most of the works, the representative quinazolinones were isolated pure without using any complex purification techniques such as column chromatography, thereby reducing the waste of solvents and time. Other remarkable properties include mild reaction conditions, broad functional group tolerances, and being environmentally benign. On the other hand, some of the existing work still needs to be developed due to the unusual requirements of longer reaction times, limited substrate scopes, and high catalyst loading.

We hope that the information presented here will stimulate and encourage the synthetic community to discover outstanding work in the elegant synthesis of these heterocycles by applying more convenient organocatalytic approaches and other synthetic approaches and to explore the potentiality of the quinazolinones to synthesize much more complex scaffolds for providing promising therapeutic significance and optoelectronic properties.

## References

[B1] Abbas S. Y. El-Bayouki K. A. Basyouni W. M. (2016). Utilization of isatoic anhydride in the syntheses of various types of quinazoline and quinazolinone derivatives. Synth. Commun. 46, 993–1035. 10.1080/00397911.2016.1177087

[B2] Anantha I. S. S. Kerru N. Maddila S. Jonnalagadda S. B. (2021). Recent progresses in the multicomponent synthesis of dihydropyridines by applying sustainable catalysts under green conditions. Front. Chem. 9, 800236. 10.3389/fchem.2021.800236 34993177 PMC8724676

[B3] Auti P. S. George G. Paul A. T. (2020). Recent advances in the pharmacological diversification of quinazoline/quinazolinone hybrids. RSC Adv. 10, 41353–41392. 10.1039/D0RA06642G 35516563 PMC9057921

[B4] Ayyanar S. Vijaya P. K. Mariyappan M. Ashokkumar V. Sadhasivam V. Balakrishnan S. (2017). Enantioselective synthesis of dihydroquinazolinone derivatives catalyzed by a chiral organocatalyst. New J. Chem. 41, 7980–7986. 10.1039/C7NJ00538E

[B5] Balalaie S. Hekmat S. Ramezanpour S. Rominger F. Kabiri-Fard H. Vavsari V. F. (2017). An environmentally friendly approach for the synthesis of quinazolinone sulfonamide. Monatsh. Chem. 148, 1453–1461. 10.1007/s00706-017-1924-x

[B6] Barbas C. F. III (2008). Organocatalysis lost: modern chemistry, ancient chemistry, and an unseen biosynthetic apparatus. Angew. Chem. Int. Ed. 47, 42–47. 10.1002/anie.200702210 17943929

[B7] Bergman J. Bergman S. (1985). Studies of rutaecarpine and related quinazolinocarboline alkaloids. J. Org. Chem. 50, 1246–1255. 10.1021/jo00208a018

[B8] Bertelsen S. Jørgensen K. A. (2009). Organocatalysis—after the gold rush. Chem. Soc. Rev. 38, 2178–2189. 10.1039/B903816G 19623342

[B9] Bhunia A. Yetra S. R. Biju A. T. (2012). Recent advances in transition-metal-free carbon–carbon and carbon–heteroatom bond-forming reactions using arynes. Chem. Soc. Rev. 41, 3140–3152. 10.1039/C2CS15310F 22278415

[B10] Borah B. Chowhan L. R. (2021). Recent advances in the transition-metal-free synthesis of quinoxalines. RSC Adv. 11, 37325–37353. 10.1039/D1RA06942J 35496411 PMC9043781

[B11] Borah B. Chowhan L. R. (2022a). Recent updates on the stereoselective synthesis of structurally functionalized spiro-oxindoles mediated by isatin N, N´ cyclic azomethine imine 1, 3-dipoles. Tetrahedron Lett. 104, 154014. 10.1016/j.tetlet.2022.154014

[B12] Borah B. Chowhan L. R. (2022b). Ultrasound-assisted transition-metal-free catalysis: a sustainable route towards the synthesis of bioactive heterocycles. RSC Adv. 12, 14022–14051. 10.1039/D2RA02063G 35558846 PMC9092113

[B13] Borah B. Dwivedi K. D. Kumar B. Chowhan L. R. (2021a). Recent advances in the microwave-and ultrasound-assisted green synthesis of coumarin-heterocycles. Arab. J. Chem. 15, 103654. 10.1016/j.arabjc.2021.103654

[B14] Borah B. Dwivedi K. D. Chowhan L. R. (2021b). Recent approaches in the organocatalytic synthesis of pyrroles. RSC Adv. 11, 13585–13601. 10.1039/D1RA01690C 35423869 PMC8697526

[B15] Borah B. Dhar Dwivedi K. Chowhan L. R. (2021c). 4‐Hydroxycoumarin: A versatile substrate for transition‐metal‐free multicomponent synthesis of bioactive heterocycles. Asian J. Org. Chem. 10, 3101–3126. 10.1002/ajoc.202100550

[B16] Borah B. Dwivedi K. D. Chowhan L. R. (2021d). Recent advances in metal‐and organocatalyzed asymmetric functionalization of pyrroles. Asian J. Org. Chem. 10, 2709–2762. 10.1002/ajoc.202100427

[B17] Borah B. Patat M. Swain S. Chowhan L. R. (2022a). Recent advances and prospects in the transition‐metal‐free synthesis of 1, 4‐dihydropyridines. ChemistrySelect 7, e202202484. 10.1002/slct.202202484

[B18] Borah B. Bora J. Ramesh P. Chowhan L. R. (2022b). Sonochemistry in an organocatalytic domino reaction: an expedient multicomponent access to structurally functionalized dihydropyrano [3, 2-b] pyrans, spiro-pyrano [3, 2-b] pyrans, and spiro-indenoquinoxaline-pyranopyrans under ambient conditions. RSC Adv. 12, 12843–12857. 10.1039/D2RA01917E 35496344 PMC9048984

[B19] Bur S. K. Padwa A. (2004). The pummerer reaction: methodology and strategy for the synthesis of heterocyclic compounds. Chem. Rev. 104, 2401–2432. 10.1021/cr020090l 15137795

[B20] Chate A. V. Rudrawar P. P. Bondle G. M. Sangeshetti J. N. (2020). 2-Aminoethanesulfonic acid: An efficient organocatalyst for green synthesis of spirooxindole dihydroquinazolinones and novel 1, 2-(dihydroquinazolin-3 (4 H) isonicotinamides in water. Synth. Commun. 50, 226–242. 10.1080/00397911.2019.1692868

[B21] Chatterjee T. Bhadra S. Ranu B. C. (2011). Transition metal-free procedure for the synthesis of S-aryl dithiocarbamates using aryl diazonium fluoroborate in water at room temperature. Green Chem. 13, 1837–1842. 10.1039/C1GC00001B

[B22] Chen A. L. Chen K. K. (1933). The constituents of Wu chü yü (Evodia rutæcarpa)**From the lilly research laboratories, eli lilly and company, indianapolis. J. Am. Pharm. Assoc. 22, 716–719. 10.1002/jps.3080220804

[B23] Chen B. H. Li J. T. Chen G. F. (2015). Efficient synthesis of 2, 3-disubstituted-2, 3-dihydroquinazolin-4 (1H)-ones catalyzed by dodecylbenzenesulfonic acid in aqueous media under ultrasound irradiation. Ultrason. Sonochem. 23, 59–65. 10.1016/j.ultsonch.2014.08.024 25224856

[B24] Chen H. Li P. Qin R. Yan H. Li G. Huang H. (2020). DMAP-catalyzed one-pot synthesis of quinazoline-2, 4-diones from 2-aminobenzamides and di-tert-butyl dicarbonate. ACS Omega 5, 9614–9623. 10.1021/acsomega.0c01104 32363314 PMC7191844

[B25] Chugh A. Kumar A. Verma A. Kumar S. Kumar P. (2020). A review of antimalarial activity of two or three nitrogen atoms containing heterocyclic compounds. Med. Chem. Res. 29, 1723–1750. 10.1007/s00044-020-02604-6

[B26] Dalko P. I. Moisan L. (2001). Enantioselective organocatalysis. Angew. Chem. Int. Ed. 40, 3726–3748. 10.1002/1521-3773(20011015)40:20<3726::AID-ANIE3726>3.0.CO;2-D 11668532

[B27] Devi J. Kalita S. J. Deka D. C. (2017). Expeditious synthesis of 2, 3-dihydroquinazolin-4 (1 H)-ones in aqueous medium using thiamine hydrochloride (VB1) as a mild, efficient, and reusable organocatalyst. Synth. Commun. 47, 1601–1609. 10.1080/00397911.2017.1337149

[B28] Dherbassy Q. Manna S. Shi C. Prasitwatcharakorn W. Crisenza G. E. Perry G. J. (2021). Enantioselective copper‐catalyzed borylative cyclization for the synthesis of quinazolinones. Angew. Chem. 133, 14476–14480. 10.1002/ange.202103259 PMC8252434 33847459

[B29] Dige N. C. Mahajan P. G. Raza H. Hassan M. Vanjare B. D. Hong H. (2019). Ultrasound mediated efficient synthesis of new 4-oxoquinazolin-3 (4H)-yl) furan-2-carboxamides as potent tyrosinase inhibitors: Mechanistic approach through chemoinformatics and molecular docking studies. Bioorg. Chem. 92, 103201. 10.1016/j.bioorg.2019.103201 31445195

[B30] D'Souza D. M. Mueller T. J. (2007). Multi-component syntheses of heterocycles by transition-metal catalysis. Chem. Soc. Rev. 36, 1095–1108. 10.1039/B608235C 17576477

[B31] Dwivedi K. D. Borah B. Chowhan L. R. (2020). Ligand free one-pot synthesis of pyrano [2, 3-c] pyrazoles in water extract of banana peel (WEB): a green chemistry approach. Front. Chem. 7, 944. 10.3389/fchem.2019.00944 32039156 PMC6987396

[B32] Fahimi N. Sardarian A. R. (2016). Citric acid: A green bioorganic catalyst for one-pot three-component synthesis of 2, 3-dihydroquinazoline-4 (1H)-ones. Curr. Organocatal. 3, 39–44. Available at: https://www.ingentaconnect.com/content/ben/cocat/2016/00000003/00000001/art00008 . 10.2174/2213337202666150602221505

[B33] Faisal M. Saeed A. (2021). Chemical insights into the synthetic chemistry of quinazolines: Recent advances. Front. Chem. 8, 594717. 10.3389/fchem.2020.594717 33585397 PMC7873916

[B34] Feng J. B. Wu X. F. (2017). Oxidative synthesis of quinazolinones under metal‐free conditions. J. Heterocycl. Chem. 54, 794–798. 10.1002/jhet.2562

[B35] Fozooni S. Firoozi H. (2015). Microwave-assisted synthesis of new quinazolinone and (dihydroquinazolinylphenyl) oxazolone derivatives. Chem. Heterocycl. Compd. (N. Y). 51, 340–345. 10.1007/s10593-015-1705-6

[B36] Gunasekara N. S. Faulds D. (1998). Raltitrexed. Drugs 55, 423–435. 10.2165/00003495-199855030-00012 9530547

[B37] Gupta T. Rohilla A. Pathak A. Akhtar M. J. Haider M. R. Yar M. S. (2018). Current perspectives on quinazolines with potent biological activities: A review. Synth. Commun. 48, 1099–1127. 10.1080/00397911.2018.1431282

[B38] Hakim F. Salfi R. Bhikshapathi D. Khan A. (2022). Anticancer evaluation of novel quinazolinone acetamides: Synthesis and characterization. Anticancer. Agents Med. Chem. 22, 926–932. 10.2174/1871520621666210524164351 34030622

[B39] Hao Y. Wang K. Wang Z. Liu Y. Ma D. Wang Q. (2020). Luotonin A and its derivatives as novel antiviral and antiphytopathogenic fungus agents. J. Agric. Food Chem. 68, 8764–8773. 10.1021/acs.jafc.0c04278 32806124

[B40] Hao S. Yang J. Liu P. Xu J. Yang C. Li F. (2021). Linear-organic-polymer-supported iridium complex as a recyclable auto-tandem catalyst for the synthesis of quinazolinones via selective hydration/acceptorless dehydrogenative coupling from o-aminobenzonitriles. Org. Lett. 23, 2553–2558. 10.1021/acs.orglett.1c00475 33729807

[B41] He L. Li H. Chen J. Wu X. F. (2014). Recent advances in 4 (3 H)-quinazolinone syntheses. RSC Adv. 4, 12065–12077. 10.1039/C4RA00351A

[B42] Hekal M. H. Abu El-Azm F. S. (2018). New potential antitumor quinazolinones derived from dynamic 2-undecyl benzoxazinone: synthesis and cytotoxic evaluation. Synth. Commun. 48, 2391–2402. 10.1080/00397911.2018.1490433

[B43] Huang H. Y. Lin X. Y. Yen S. Y. Liang C. F. (2020). Facile access to N-formyl imide as an N-formylating agent for the direct synthesis of N-formamides, benzimidazoles and quinazolinones. Org. Biomol. Chem. 18, 5726–5733. 10.1039/D0OB01080D 32666985

[B44] Jia F. C. Chen T. Z. Hu X. Q. (2020). TFA/TBHP-promoted oxidative cyclisation for the construction of tetracyclic quinazolinones and rutaecarpine. Org. Chem. Front. 7, 1635–1639. 10.1039/D0QO00345J

[B45] Karhale S. Survase D. Bhat R. Ubale P. Helavi V. (2017). A practical and green protocol for the synthesis of 2, 3-dihydroquinazolin-4 (1H)-ones using oxalic acid as organocatalyst. Res. Chem. Intermed. 43, 3915–3924. 10.1007/s11164-016-2855-6

[B46] Kawade D. S. Chaudhari M. A. Gujar J. B. Shingare M. S. (2016). Thiamine hydrochloride (vitamin B1) as an efficient catalyst for the synthesis of 4-(3H)-Quinazolinone derivatives using grinding method. Iran. J. Catal. 6, 313–318.

[B47] Khan I. Ibrar A. Abbas N. Saeed A. (2014). Recent advances in the structural library of functionalized quinazoline and quinazolinone scaffolds: Synthetic approaches and multifarious applications. Eur. J. Med. Chem. 76, 193–244. 10.1016/j.ejmech.2014.02.005 24583357

[B48] Khan I. Ibrar A. Ahmed W. Saeed A. (2015). Synthetic approaches, functionalization and therapeutic potential of quinazoline and quinazolinone skeletons: the advances continue. Eur. J. Med. Chem. 90, 124–169. 10.1016/j.ejmech.2014.10.084 25461317

[B49] Khan I. Zaib S. Batool S. Abbas N. Ashraf Z. Iqbal J. (2016). Quinazolines and quinazolinones as ubiquitous structural fragments in medicinal chemistry: An update on the development of synthetic methods and pharmacological diversification. Bioorg. Med. Chem. 24, 2361–2381. 10.1016/j.bmc.2016.03.031 27112448

[B50] Khan I. Zaib S. Ibrar A. (2020). New frontiers in the transition-metal-free synthesis of heterocycles from alkynoates: an overview and current status. Org. Chem. Front. 7, 3734–3791. 10.1039/D0QO00698J

[B51] Khandebharad A. U. Sarda S. R. Gill C. H. Agrawal B. R. (2020). Synthesis of quinazolinone derivatives catalyzed by triethanolamine/NaCl in aqueous media. Polycycl. Aromat. Compd. 40, 437–445. 10.1080/10406638.2018.1441884

[B52] Köhne C. H. Thuss-Patience P. Friedrich M. Daniel P. T. Kretzschmar A. Benter T. (1998). Raltitrexed (Tomudex): an alternative drug for patients with colorectal cancer and 5-fluorouracil associated cardiotoxicity. Br. J. Cancer 77, 973–977. 10.1038/bjc.1998.160 9528843 PMC2150099

[B53] Kshirsagar U. A. (2015). Recent developments in the chemistry of quinazolinone alkaloids. Org. Biomol. Chem. 13, 9336–9352. 10.1039/C5OB01379H 26278395

[B54] Kumar B. Babu N. J. Chowhan R. L. (2022). Sustainable synthesis of highly diastereoselective & fluorescent active spirooxindoles catalyzed by copper oxide nanoparticle immobilized on microcrystalline cellulose. Appl. Organomet. Chem. 36, e6742. 10.1002/aoc.6742

[B55] Kumar P. Tomar V. Joshi R. K. Nemiwal M. (2022). Nanocatalyzed synthetic approach for quinazoline and quinazolinone derivatives: A review (2015–present). Synth. Commun. 52, 795–826. 10.1080/00397911.2022.2041667

[B56] Lee S. Sim J. Jo H. Viji M. Srinu L. Lee K. (2019). Transition metal-free synthesis of quinazolinones using dimethyl sulfoxide as a synthon. Org. Biomol. Chem. 17, 8067–8070. 10.1039/C9OB01629E 31451812

[B57] Li Z. Dong J. Chen X. Li Q. Zhou Y. Yin S. F. (2015). Metal-and oxidant-free synthesis of quinazolinones from β-ketoesters with o-aminobenzamides via phosphorous acid-catalyzed cyclocondensation and selective C–C bond cleavage. J. Org. Chem. 80, 9392–9400. 10.1021/acs.joc.5b00937 26339716

[B58] Lin Y. He S. F. Geng H. Xiao Y. C. Ji K. L. Zheng J. F. (2021). Chemoselective reactions of isocyanates with secondary amides: One-pot construction of 2, 3-dialkyl-substituted quinazolinones. J. Org. Chem. 86, 5345–5353. 10.1021/acs.joc.0c02929 33710879

[B59] List B. (2007). Introduction: organocatalysis. Chem. Rev. 107, 5413–5415. 10.1021/cr078412e

[B60] Liu H. Zhai T. Ding S. Hou Y. Zhang X. Feng L. (2016). Direct and metal-free oxidative amination of sp 3 C–H bonds for the construction of 2-hetarylquinazolin-4 (3 H)-ones. Org. Chem. Front. 3, 1096–1099. 10.1039/C6QO00231E

[B61] Ma Z. Z. Hano Y. Nomura T. Chen Y. J. (1997). Two new pyrroloquinazolinoquinoline alkaloids from Peganum nigellastrum. Heterocycles 46, 541–546. 10.3987/com-97-s65

[B62] MacMillan D. W. (2008). The advent and development of organocatalysis. Nature 455 (7211), 304–308. 10.1038/nature07367 18800128

[B63] Maiden T. M. M. Harrity J. P. A. (2016). Recent developments in transition metal catalysis for quinazolinone synthesis. Org. Biomol. Chem. 14, 8014–8025. 10.1039/C6OB01402J 27477737

[B64] Majumdar P. Pati A. Patra M. Behera R. K. Behera A. K. (2014). Acid hydrazides, potent reagents for synthesis of oxygen-nitrogen-and/or sulfur-containing heterocyclic rings. Chem. Rev. 114, 2942–2977. 10.1021/cr300122t 24506477

[B65] Mehta H. B. Dixit B. C. Dixit R. B. (2014). L-Proline catalyzed one-pot multi-component synthesis of 2-(1, 3-diphenyl-1H-pyrazol-4-yl) quinazolin-4 (3H)-one derivatives and their biological studies. Chin. Chem. Lett. 25, 741–744. 10.1016/j.cclet.2014.03.015

[B66] Mermer A. Keles T. Sirin Y. (2021). Recent studies of nitrogen containing heterocyclic compounds as novel antiviral agents: A review. Bioorg. Chem. 114, 105076. 10.1016/j.bioorg.2021.105076 34157555

[B67] Mhaske S. B. Argade N. P. (2006). The chemistry of recently isolated naturally occurring quinazolinone alkaloids. Tetrahedron 62, 9787–9826. 10.1016/j.tet.2006.07.098

[B68] Mohammadkhani L. Heravi M. M. (2020). Microwave-assisted synthesis of quinazolines and quinazolinones: an overview. Front. Chem. 8, 580086. 10.3389/fchem.2020.580086 33282829 PMC7705381

[B69] Moon T. C. Murakami M. Kudo I. Son K. H. Kim H. P. Kang S. S. (1999). A new class of COX-2 inhibitor, rutaecarpine from Evodia rutaecarpa. Inflamm. Res. 48, 621–625. 10.1007/s000110050512 10669112

[B70] Nakamura I. Yamamoto Y. (2004). Transition-metal-catalyzed reactions in heterocyclic synthesis. Chem. Rev. 104, 2127–2198. 10.1021/cr020095i 15137788

[B71] Patrusheva O. S. Volcho K. P. Salakhutdinov N. F. (2018). Synthesis of oxygen-containing heterocyclic compounds based on monoterpenoids. Russ. Chem. Rev. 87, 771–796. 10.1070/RCR4810

[B72] Peng J. Lin T. Wang W. Xin Z. Zhu T. Gu Q. (2013). Antiviral alkaloids produced by the mangrove-derived fungus Cladosporium sp. PJX-41. J. Nat. Prod. 76, 1133–1140. 10.1021/np400200k 23758051

[B73] Phakhodee W. Wangngae S. Pattarawarapan M. (2017). Approach to the synthesis of 2, 3-disubstituted-3 H-quinazolin-4-ones mediated by Ph3P–I2. J. Org. Chem. 82, 8058–8066. 10.1021/acs.joc.7b01322 28721733

[B74] Poudel T. N. Tamargo R. J. I. Cai H. Lee Y. R. (2018). Recent progress in transition‐metal‐free, base‐mediated benzannulation reactions for the synthesis of a diverse range of aromatic and heteroaromatic compounds. Asian J. Org. Chem. 7, 985–1005. 10.1002/ajoc.201800080

[B75] Rahman M. Ling I. Abdullah N. Hashim R. Hajra A. (2015). Organocatalysis by p-sulfonic acid calix [4] arene: a convenient and efficient route to 2, 3-dihydroquinazolin-4 (1 H)-ones in water. RSC Adv. 5, 7755–7760. 10.1039/C4RA16374E

[B76] Ramesh R. Kalisamy P. Malecki J. G. Lalitha A. (2018). Metal-free mild synthesis of novel 1′ H-spiro [cycloalkyl-1, 2′-quinazolin]-4′(3′ H)-ones by an organocatalytic cascade reaction. Synlett 29, 203–208. 10.1055/s-0036-1590917

[B77] Rao Y. Liu H. Gao L. Yu H. Tan J. H. Ou T. M. (2015). Discovery of natural alkaloid bouchardatine as a novel inhibitor of adipogenesis/lipogenesis in 3T3-L1 adipocytes. Bioorg. Med. Chem. 23, 4719–4727. 10.1016/j.bmc.2015.05.057 26088335

[B78] Reetz M. T. (2013). Biocatalysis in organic chemistry and biotechnology: past, present, and future. J. Am. Chem. Soc. 135, 12480–12496. 10.1021/ja405051f 23930719

[B79] Ren Q. Wang J. (2013). Recent developments in amine‐catalyzed non‐asymmetric transformations. Asian J. Org. Chem. 2, 542–557. 10.1002/ajoc.201200191

[B80] Renzi P. Bella M. (2012). Non-asymmetric organocatalysis. Chem. Commun. 48, 6881–6896. 10.1039/C2CC31599H 22662324

[B81] Revathy K. Lalitha A. (2015). p-TSA-catalyzed synthesis of spiroquinazolinones. J. Iran. Chem. Soc. 12, 2045–2049. 10.1007/s13738-015-0680-2

[B82] Rohokale R. S. Kshirsagar U. A. (2016). Advanced synthetic strategies for constructing quinazolinone scaffolds. Synthesis 48, 1253–1268. 10.1055/s-0035-1560413

[B83] Rotstein B. H. Zaretsky S. Rai V. Yudin A. K. (2014). Small heterocycles in multicomponent reactions. Chem. Rev. 114, 8323–8359. 10.1021/cr400615v 25032909

[B84] Shanafelt T. D. Borah B. J. Finnes H. D. Chaffee K. G. Ding W. Leis J. F. (2015). Impact of ibrutinib and idelalisib on the pharmaceutical cost of treating chronic lymphocytic leukemia at the individual and societal levels. J. Oncol. Pract. 11, 252–258. 10.1200/JOP.2014.002469 25804983

[B85] Shang X. Liu Z. Q. (2013). Transition metal-catalyzed C vinyl–C vinyl bond formation via double C vinyl–H bond activation. Chem. Soc. Rev. 42, 3253–3260. 10.1039/C2CS35445D 23318664

[B86] Shen G. Zhou H. Du P. Liu S. Zou K. Uozumi Y. (2015). Brønsted acid-catalyzed selective C–C bond cleavage of 1, 3-diketones: a facile synthesis of 4 (3 H)-quinazolinones in aqueous ethyl lactate. RSC Adv. 5, 85646–85651. 10.1039/C5RA17969F

[B87] Sugai T. Yamazaki T. Yokoyama M. Ohta H. (1997). Biocatalysis in organic synthesis: the use of nitrile-and amide-hydrolyzing microorganisms. Biosci. Biotechnol. Biochem. 61, 1419–1427. 10.1271/bbb.61.1419

[B88] Sun C. L. Shi Z. J. (2014). Transition-metal-free coupling reactions. Chem. Rev. 114, 9219–9280. 10.1021/cr400274j 25184859

[B89] Wang Y. B. Zheng S. C. Hu Y. M. Tan B. (2017). Brønsted acid-catalysed enantioselective construction of axially chiral arylquinazolinones. Nat. Commun. 8, 15489. 10.1038/ncomms15489 28524863 PMC5454535

[B90] Xing Z. Wu W. Miao Y. Tang Y. Zhou Y. Zheng L. (2021). Recent advances in quinazolinones as an emerging molecular platform for luminescent materials and bioimaging. Org. Chem. Front. 8, 1867–1889. 10.1039/D0QO01425G

[B91] Yang X. Cheng G. Shen J. Kuai C. Cui X. (2015). Cleavage of the C–C triple bond of ketoalkynes: synthesis of 4 (3 H)-quinazolinones. Org. Chem. Front. 2, 366–368. 10.1039/C4QO00260A

[B92] Yashwantrao G. Jejurkar V. P. Kshatriya R. Saha S. (2019). Solvent-free, mechanochemically scalable synthesis of 2, 3-dihydroquinazolin-4 (1H)-one using Brønsted acid catalyst. ACS Sustain. Chem. Eng. 7, 13551–13558. 10.1021/acssuschemeng.9b03199

[B93] Zhou Q. L. (2016). Transition‐metal catalysis and organocatalysis: where can progress be expected? Angew. Chem. Int. Ed. 55, 5352–5353. 10.1002/anie.201509164 26662619

[B94] Zirlik K. Veelken H. (2018). “Idelalisib,” in Small molecules in hematology. Editor Martens U. (Cham: Springer), Recent Results in Cancer Research. Vol. 212, 243–264. 10.1007/978-3-319-91439-8_12 30069634

